# Supplementary material for: A Flexible Tool to Correct Superimposed Mass Isotopologue Distributions in GC-APCI-MS Flux Experiments
Source: Metabolites. 2022 Apr 29;12(5):408. doi: 10.3390/metabo12050408 (PMC9144802; doi:10.3390/metabo12050408)
Supplement: Supplementary file 1 [file metabolites-12-00408-s001.zip › metabolites-1680326-supplementary.pdf]

metabolites

Supporting Information: A flexible tool to correct superimposed mass isotopologue distributions in GC-APCI-MS flux experiments

Jennifer Langenhan [1], Carsten Jaeger [1], Katharina Baum [2], Mareike Simon [2], and Jan Lisec [1,*]

[1] Bundesanstalt für Materialforschung und -prüfung (BAM), Division 1 Analytical Chemistry, Richard-Willstätter-Straße 11, 12489 Berlin, Germany
[2] Max-Delbrück-Center for Molecular Medicine (MDC), Mathematical Modeling of Cellular Processes, Robert-Rössle-Straße 10, 13125 Berlin, Germany
[3] Hasso Plattner Institute, Digital Engineering Faculty, University of Potsdam, Prof.-Dr.-Helmert-Straße 2-3, 14482 Potsdam, Germany
* Correspondence: jan.lisec@bam.de; Tel.: 0049 (0)30 8104 5891

Table S1. Comparison of features of IsoCor, MIDcor, IsoCorrectoR, and CorMID.

Corrects for:	IsoCor [12]	MIDcor [10]	IsoCorrectoR [11]	CorMID
natural abundance	x	x	x	x
tracer purity			x	
overlap with other Compounds		x		
proton loss		x		x
overlap with other in-source fragments				x
overlap with rearrangement products				x

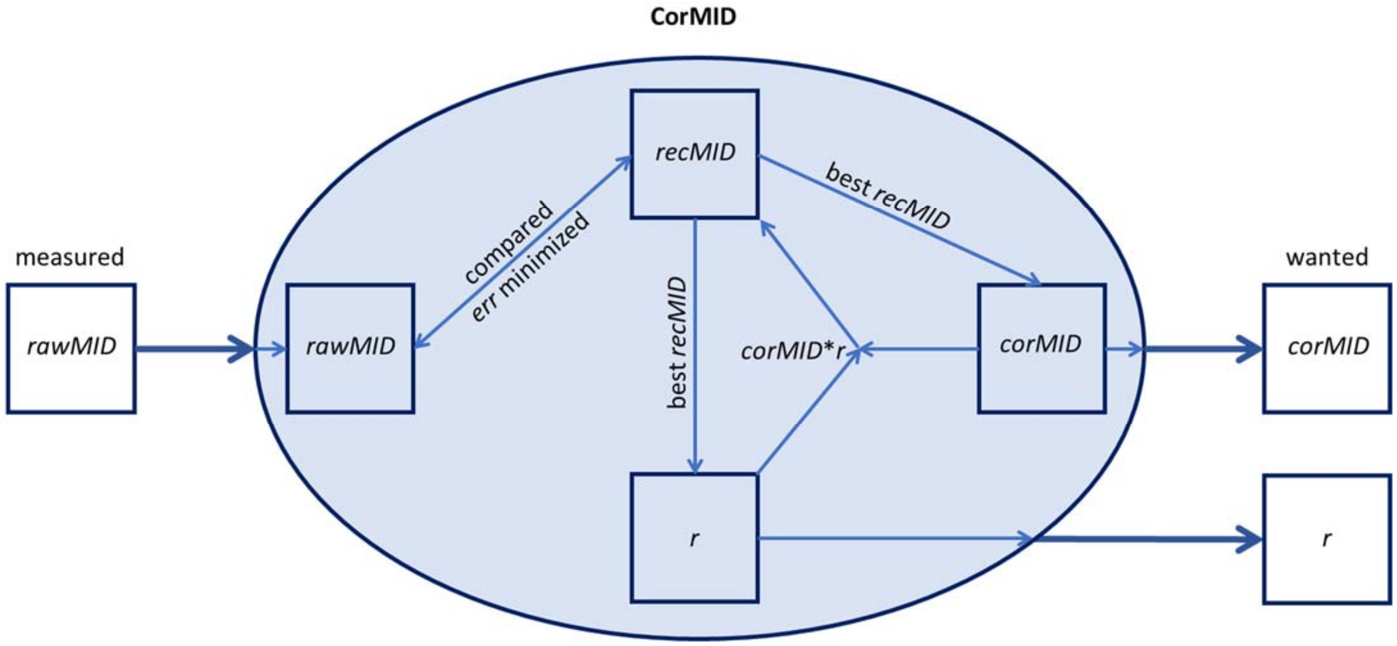

Figure S1. The function CorMID works as follows: a residual error (*err*) between an observed vector of measured ion intensities (*rawMID*) and a reconstructed vector of similar size (*recMID*) is calculated out of a fragment distribution vector (*r*) and the corrected MID (*corMID*). The size of *r* is dependent on the number of fragments considered and the size of vector *corMID* is dependent on the number carbon atoms within the molecule. Under normal operating conditions *r* and *corMID* are estimated until the best *recMID* (*err* minimized) is found. *r* or *corMID* can also be manually fixed. That leads to a unique solution of the function. The *corMID* and *r*, which lead to the best *recMID* are the output of CorMID.

Figure S2. Evaluation of library compound 22, 3-DEHYDROSHIKIMATE (1MeOx, 3TMS). (A) The measured intensities of the compound are normalized to the vector sum. The main adduct $[M+H]^+$ is represented as M+0 in the spectrum. The true MID (*corMID*) and fragment distribution *r* are unknown, which is indicated by two question marks. (B) Assuming no artificial labelling we can estimate the fragment distribution which fits the observed data best and use this *r* to reconstruct the expected measurement values. The error between the reconstructed MID and the measured MID is annotated in the spectrum. (C) Using *r* as obtained in (B) as a fixed parameter and estimating the optimal MID, we observe a much better fit of the measured data when assuming approximately 23% M1 labelling. (D) For comparison we can estimate MID and *r* in parallel which further reduces the fitting error in the reconstructed MID. In conclusion, this example shows that dubious peak intensities, i.e. as a result of impurities, will hamper the correct estimation of MID and *r*. In the example the M+1 is most likely wrong and shows too high intensity. We found such problems in approximately 10% of all library samples.

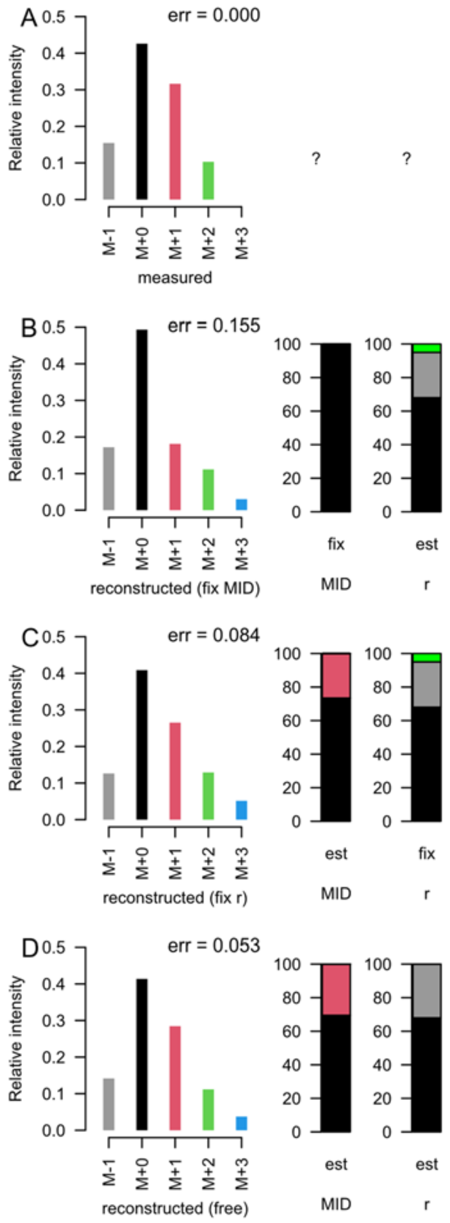

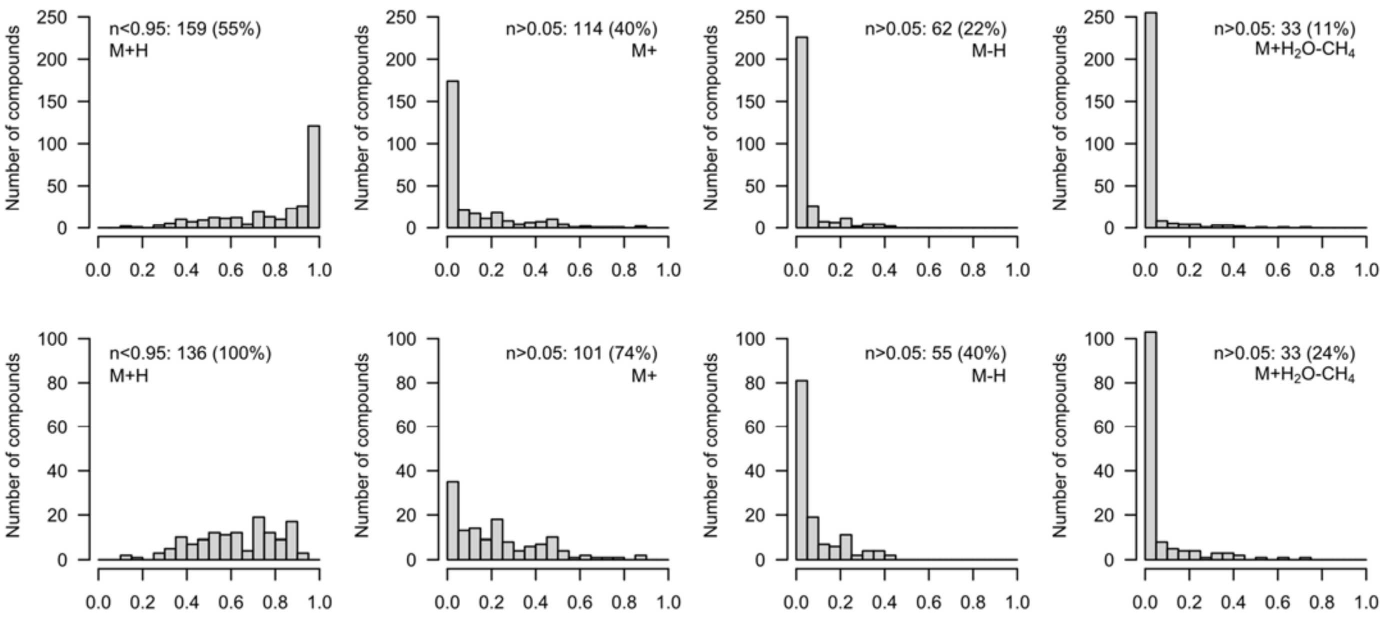

Figure S3. Distribution of calculated fragment fractions for all 288 compounds with M0=100% (top row, similar to Figure 3). For comparison we estimated *corMID* using a fixed *r* (with $[M+H]^+$ =1) to find the number of compounds that show >5% deviation from the correct M0=100% without considering fragments other than $[M+H]^+$. The r for this subset of 136 compounds is depicted in the bottom row. All 33 compounds which showed the fragment $[M+H3O-CH4]^+$ did yield a wrong M0 when the fragment was not considered. The same was true for most of the $[M]^+$ (101 of 114) and $[M-H]^+$ (55 of 62) fragments.

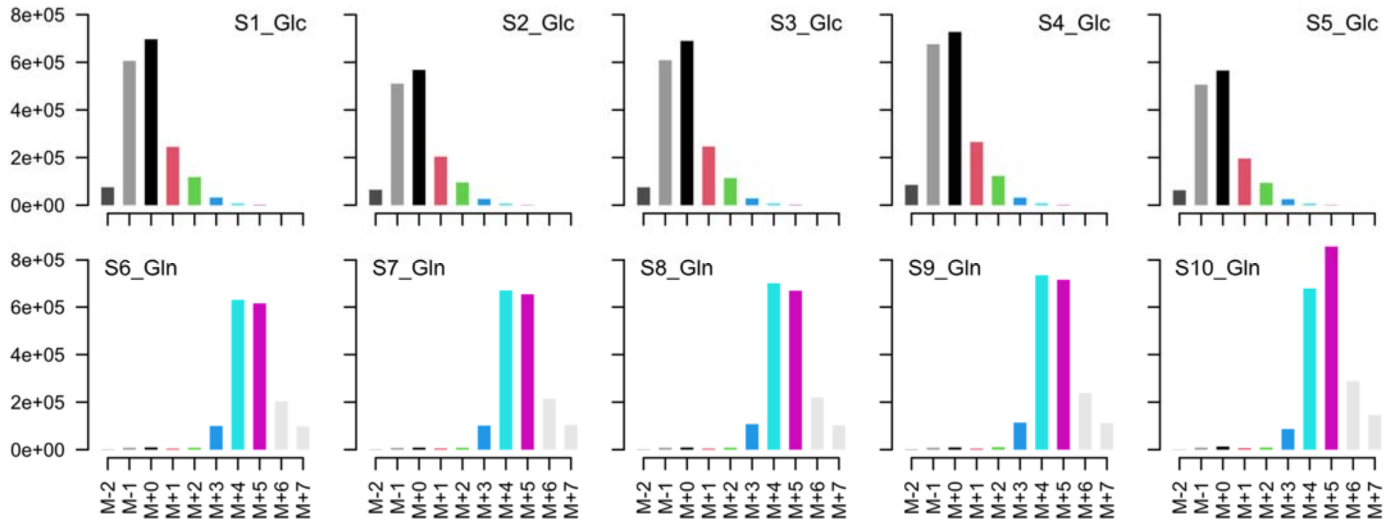

Figure S4. *rawMID* intensities of Gln after Glc labeling (upper row), *rawMID* intensities of Gln after Gln labeling (bottom row). Notice the intensity difference for the fifth replicate, leading to a different MID and fragment distribution see Figure 5A-B.

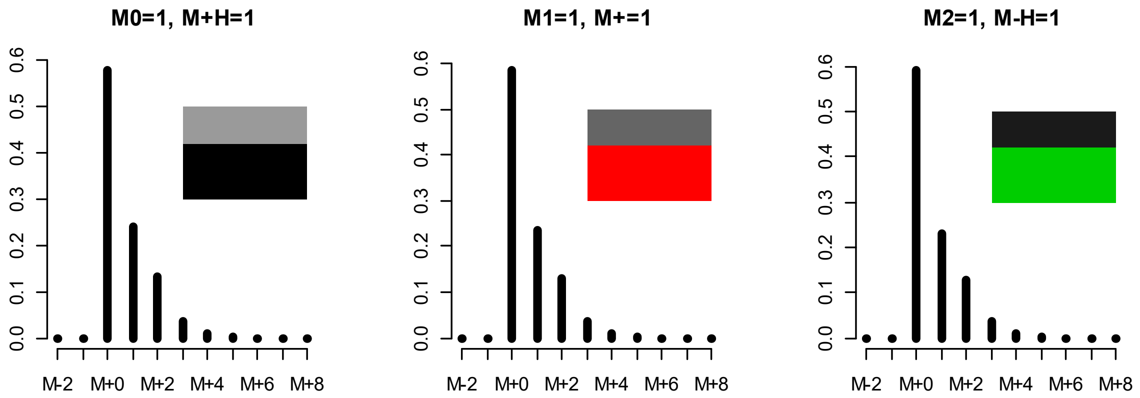

Figure S5. Three reconstructed *rawMIDs* (equivalent to normalized measured ion intensities) based on MID and fragment distribution as presented in the figure title. Spectra are identical within the limits of APCI-MS (error ~2% deviation).

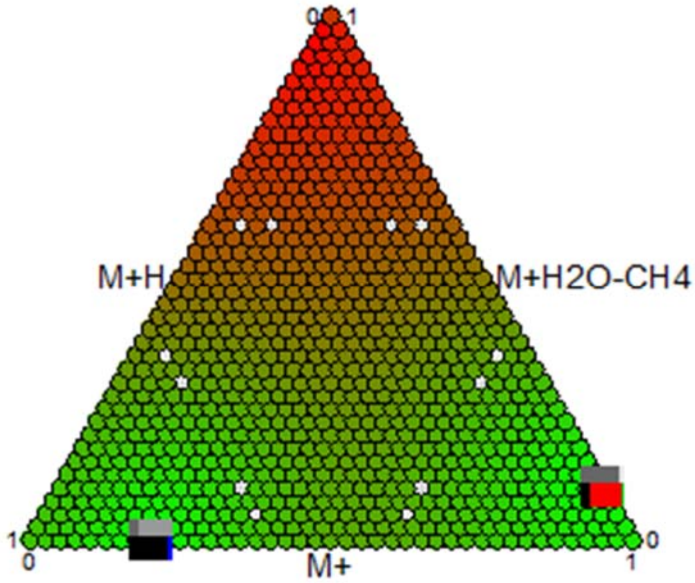

Figure S6. Heuristic error distribution of free fit (i.e. estimating *corMID* and fragment distribution at the same time) for 20% M3 pyruvic acid with 10% $[M]^+$. Each circle within the triangle defines a unique combination of *r* (fragment distribution), allowing here only 3 fragments to facilitate visualization. The color of each circle indicates the smallest fitting error achievable for this *r*. Without any weighting two equivalent local minima exist in the solution space.

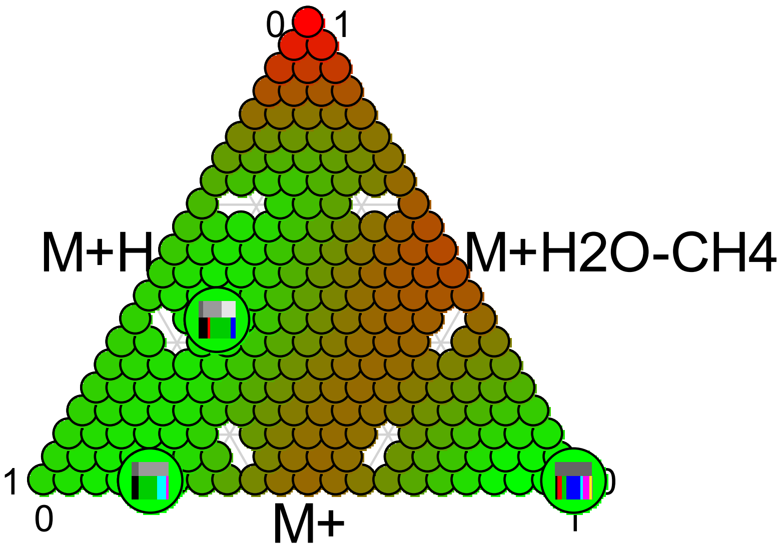

Figure S7. Heuristic error distribution of free fit (i.e. estimating MID and fragment distribution at the same time) for Citrulline with MID={0.2,0,0.5,0,0.25,0.05,0} and fragment distribution={0.8 $[M+H]^+$, 0.2 $[M]^+$}. Without any weighting three local minima exist in the solution space.
