# Supplementary material for: A Flexible Tool to Correct Superimposed Mass Isotopologue Distributions in GC-APCI-MS Flux Experiments
Source: Metabolites. 2022 Apr 29;12(5):408. doi: 10.3390/metabo12050408 (PMC9144802; doi:10.3390/metabo12050408)
Supplement: Supplementary file 1 [file metabolites-12-00408-s001.zip › SI-File S1.pdf]

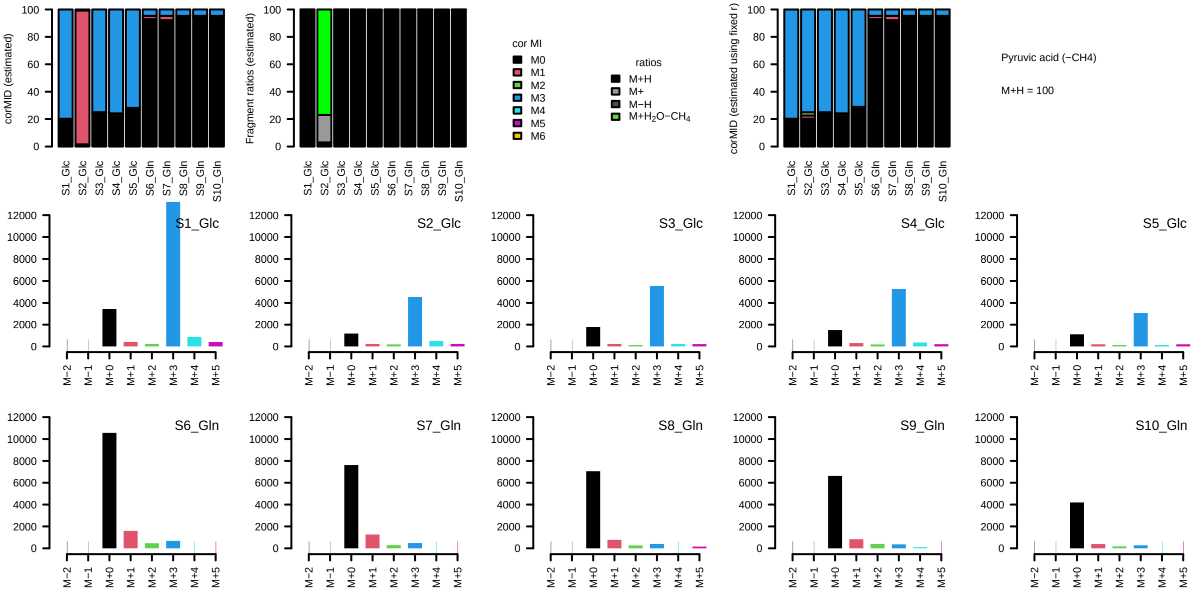

corMID (estimated)
Fragment ratios (estimated)
corMID (estimated using fixed r)
cor MI
M0
M1
M2
M3
M4
M5
M6
ratios
M+H
M+
M−H
M+H2O−CH4
Pyruvic acid (−CH4)
M+H = 100
S1_Glc
S2_Glc
S3_Glc
S4_Glc
S5_Glc
S6_Gln
S7_Gln
S8_Gln
S9_Gln
S10_Gln
M−2
M−1
M+0
M+1
M+2
M+3
M+4
M+5

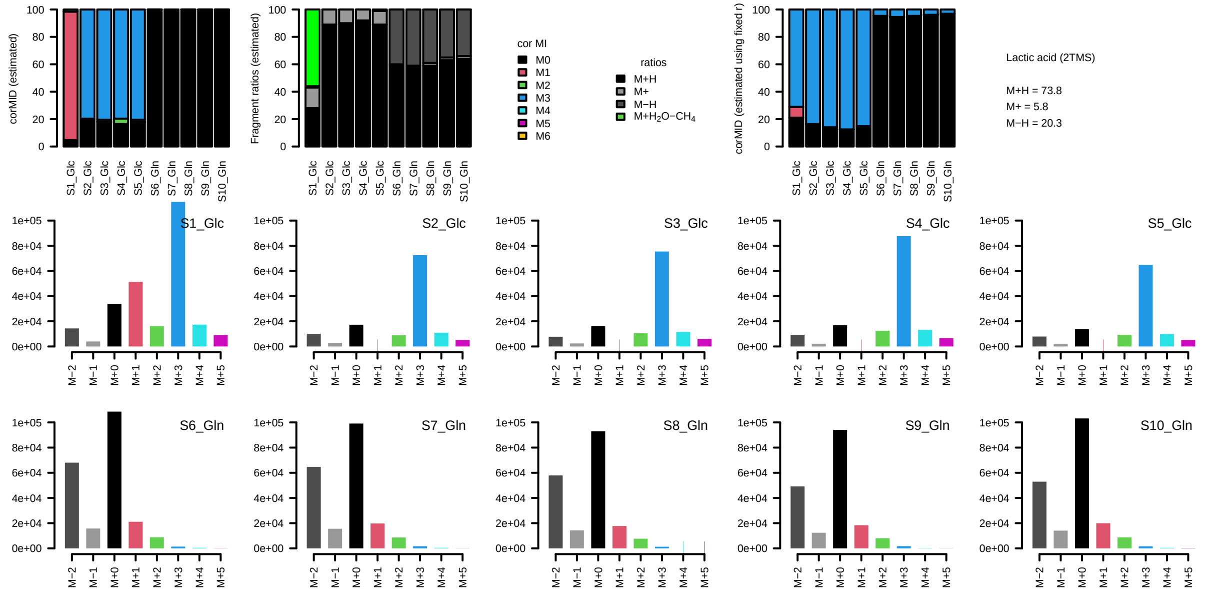

corMID (estimated)
Fragment ratios (estimated)
corMID (estimated using fixed r)
cor MI
M0
M1
M2
M3
M4
M5
M6
ratios
M+H
M+
M−H
M+H2O−CH4
Lactic acid (2TMS)
M+H = 73.8
M+ = 5.8
M−H = 20.3
S1_Glc
S2_Glc
S3_Glc
S4_Glc
S5_Glc
S6_Gln
S7_Gln
S8_Gln
S9_Gln
S10_Gln
M−2
M−1
M+0
M+1
M+2
M+3
M+4
M+5

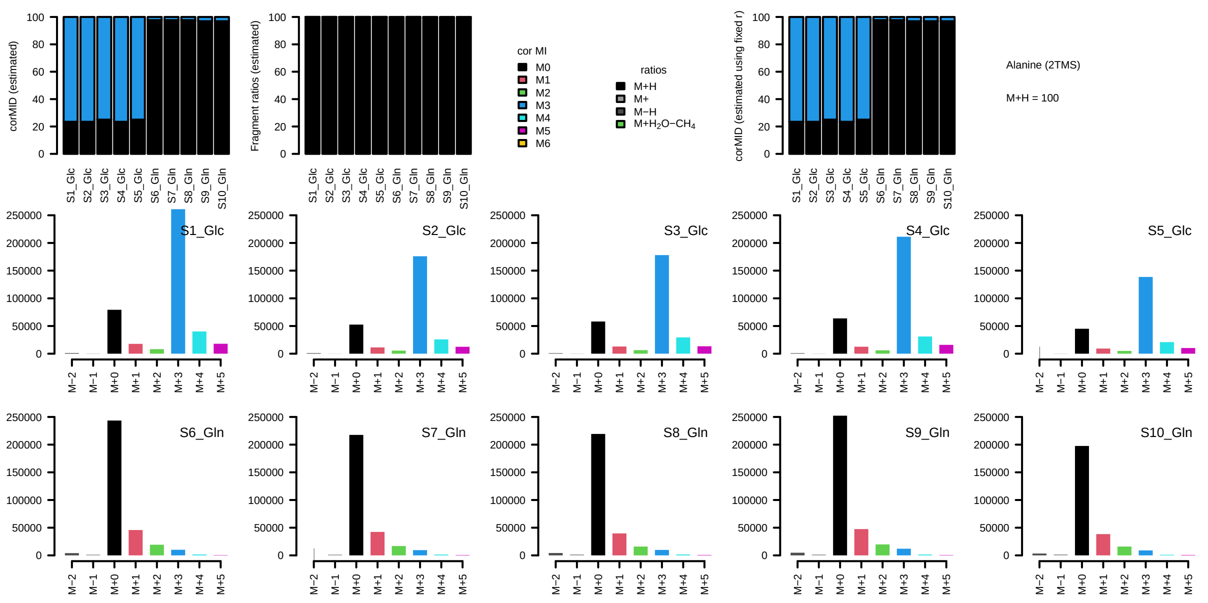
corMID (estimated)
Fragment ratios (estimated)
corMID (estimated using fixed r)
S1_Glc
S2_Glc
S3_Glc
S4_Glc
S5_Glc
S6_Gln
S7_Gln
S8_Gln
S9_Gln
S10_Gln
cor MI
M0
M1
M2
M3
M4
M5
M6
ratios
M+H
M+
M−H
M+H2O−CH4
Alanine (2TMS)
M+H = 100
M−2
M−1
M+0
M+1
M+2
M+3
M+4
M+5

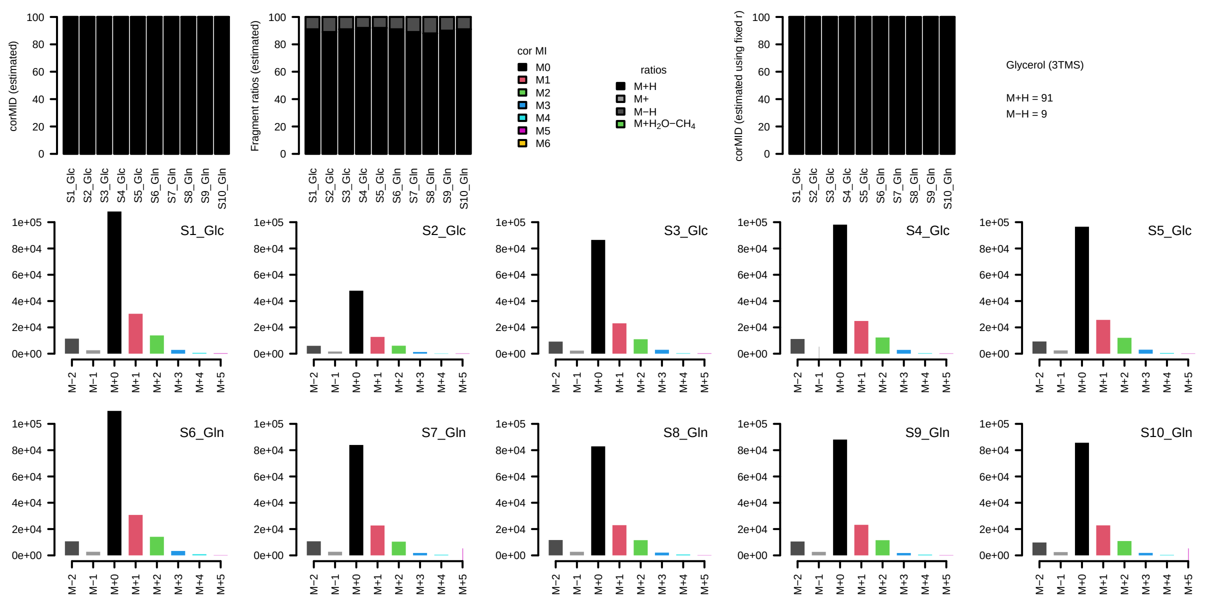
corMID (estimated)
Fragment ratios (estimated)
cor MI
M0
M1
M2
M3
M4
M5
M6
ratios
M+H
M+
M−H
M+H_2O−CH_4
corMID (estimated using fixed r)
Glycerol (3TMS)
M+H = 91
M−H = 9
S1_Glc
S2_Glc
S3_Glc
S4_Glc
S5_Glc
S6_Gln
S7_Gln
S8_Gln
S9_Gln
S10_Gln
M−2
M−1
M+0
M+1
M+2
M+3
M+4
M+5

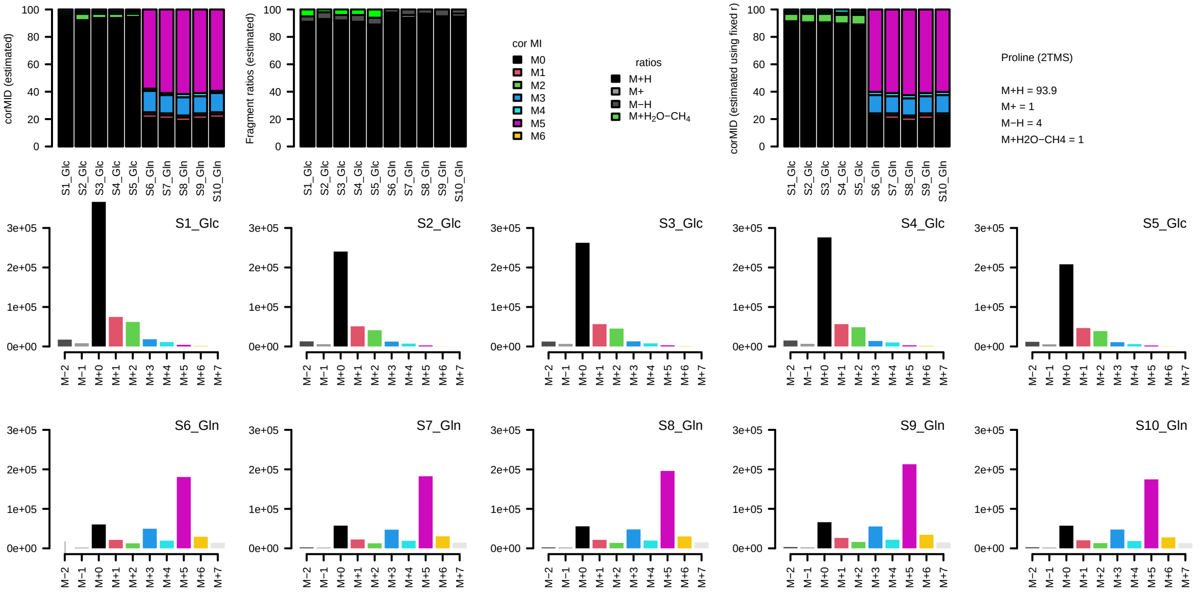

corMID (estimated)
Fragment ratios (estimated)
corMID (estimated using fixed r)
S1_Glc S2_Glc S3_Glc S4_Glc S5_Glc S6_Gln S7_Gln S8_Gln S9_Gln S10_Gln
cor MI
M0
M1
M2
M3
M4
M5
M6
ratios
M+H
M+
M−H
M+H$_2$O−CH$_4$
Proline (2TMS)
M+H = 93.9
M+ = 1
M−H = 4
M+H2O−CH4 = 1
S1_Glc
S2_Glc
S3_Glc
S4_Glc
S5_Glc
S6_Gln
S7_Gln
S8_Gln
S9_Gln
S10_Gln
M−2 M−1 M+0 M+1 M+2 M+3 M+4 M+5 M+6 M+7

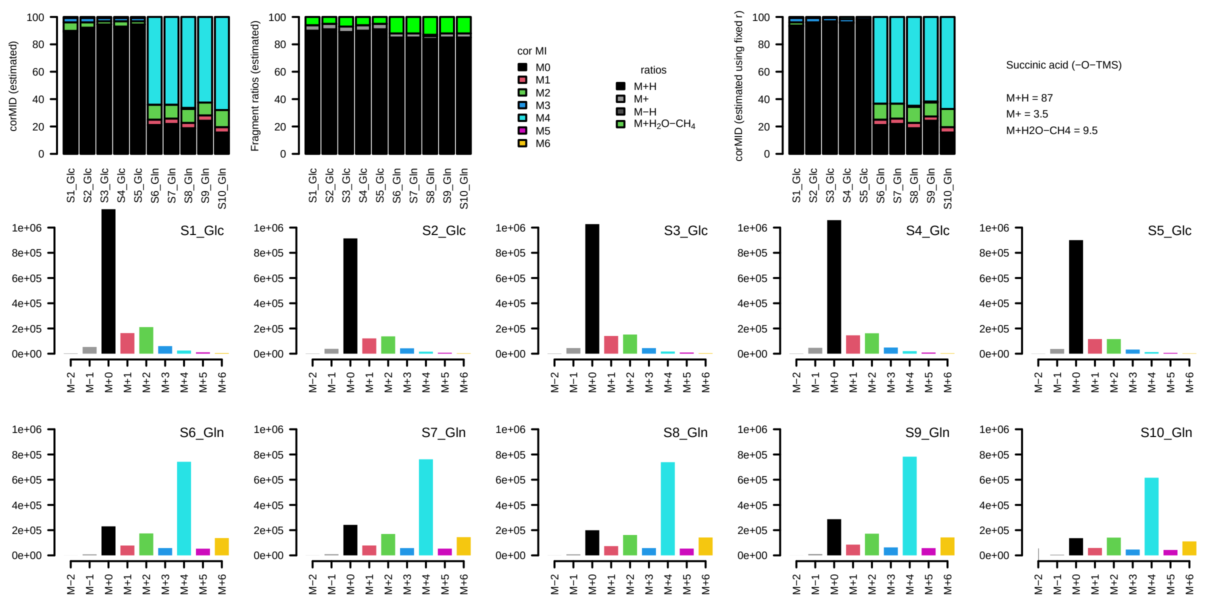
corMID (estimated)
Fragment ratios (estimated)
corMID (estimated using fixed r)
cor MI
M0
M1
M2
M3
M4
M5
M6
ratios
M+H
M+
M−H
M+H$_2$O−CH$_4$
Succinic acid (−O−TMS)
M+H = 87
M+ = 3.5
M+H2O−CH4 = 9.5
S1_Glc
S2_Glc
S3_Glc
S4_Glc
S5_Glc
S6_Gln
S7_Gln
S8_Gln
S9_Gln
S10_Gln
M−2
M−1
M+0
M+1
M+2
M+3
M+4
M+5
M+6

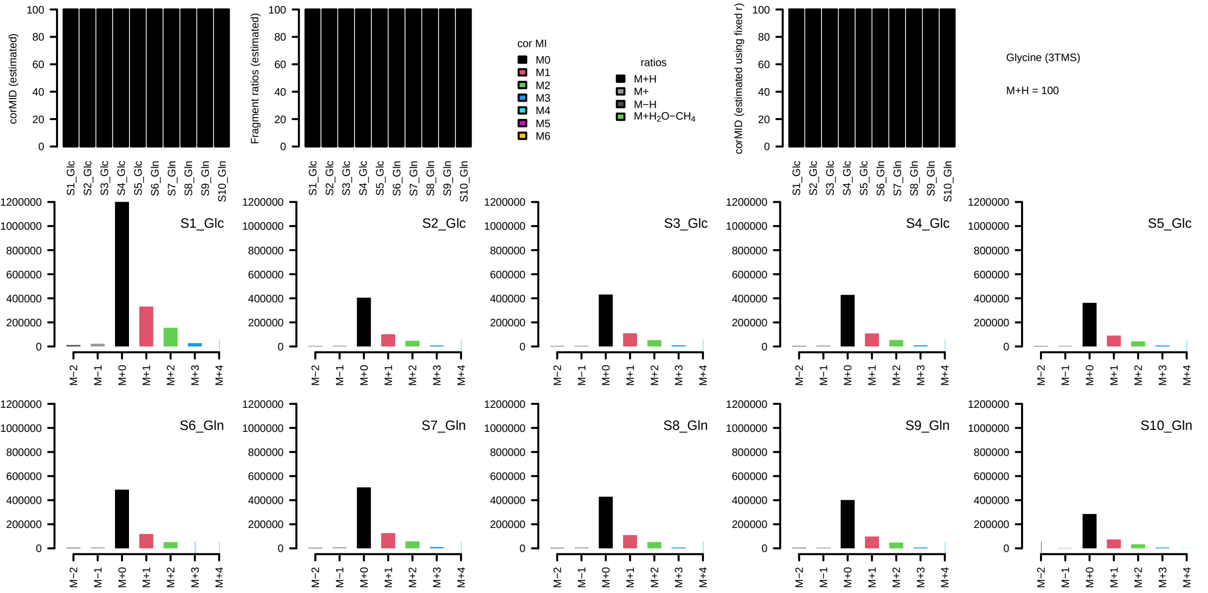
corMID (estimated)
Fragment ratios (estimated)
cor MI
M0
M1
M2
M3
M4
M5
M6
ratios
M+H
M+
M−H
M+H2O−CH4
corMID (estimated using fixed r)
Glycine (3TMS)
M+H = 100
S1_Glc
S2_Glc
S3_Glc
S4_Glc
S5_Glc
S6_Gln
S7_Gln
S8_Gln
S9_Gln
S10_Gln
M−2
M−1
M+0
M+1
M+2
M+3
M+4

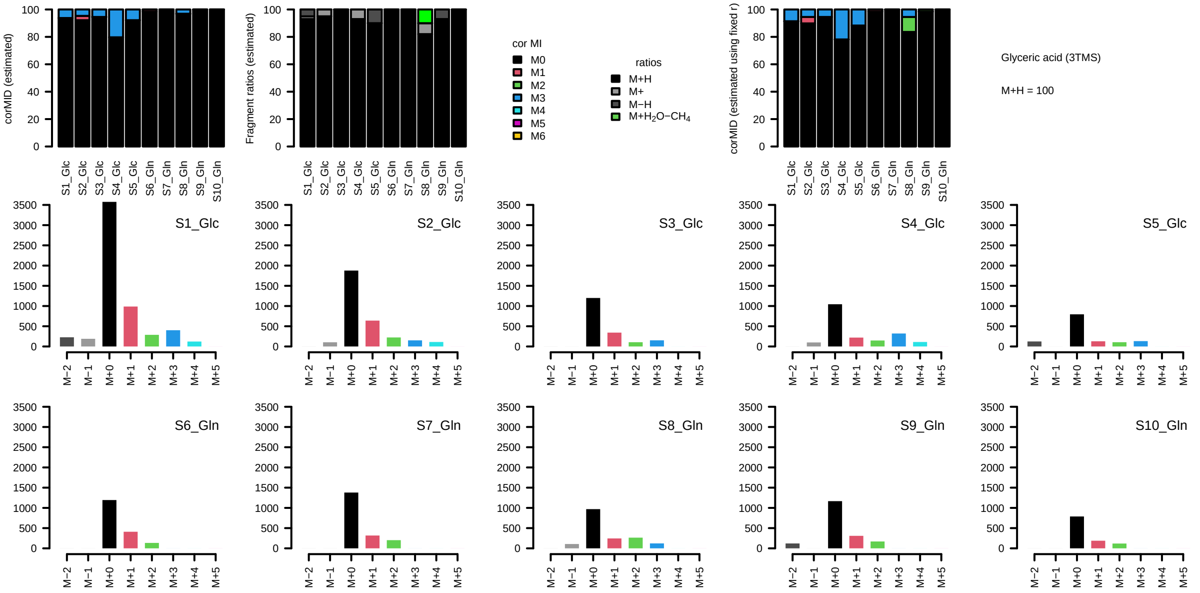

corMID (estimated)
Fragment ratios (estimated)
cor MI
M0
M1
M2
M3
M4
M5
M6
ratios
M+H
M+
M−H
M+H2O−CH4
corMID (estimated using fixed r)
Glyceric acid (3TMS)
M+H = 100
S1_Glc
S2_Glc
S3_Glc
S4_Glc
S5_Glc
S6_Gln
S7_Gln
S8_Gln
S9_Gln
S10_Gln
M−2
M−1
M+0
M+1
M+2
M+3
M+4
M+5

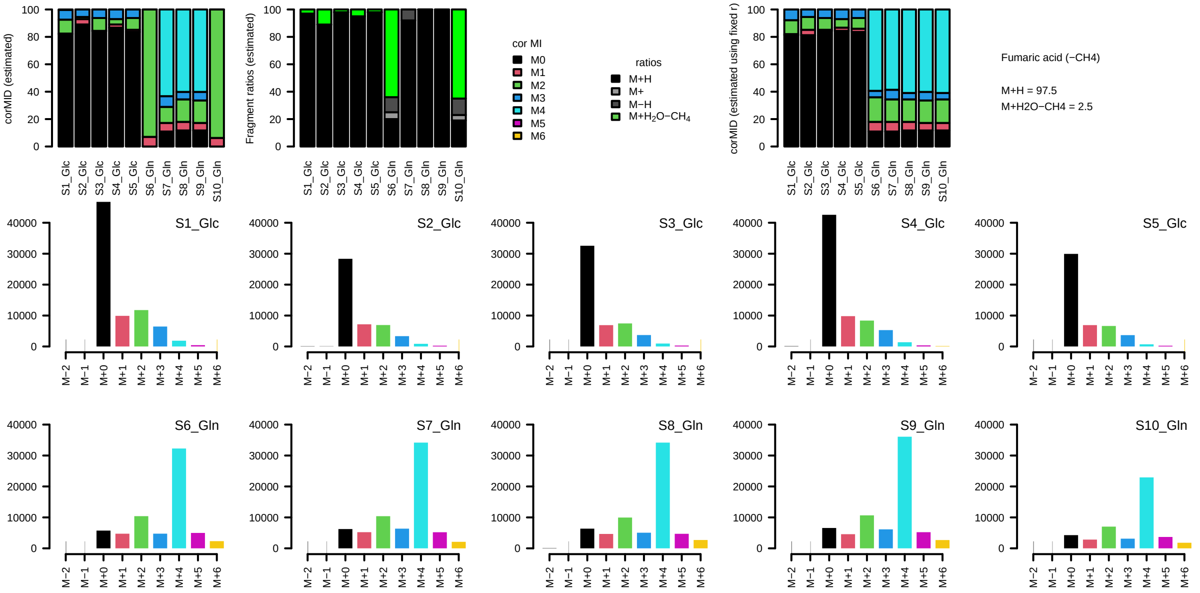

corMID (estimated)
Fragment ratios (estimated)
corMID (estimated using fixed r)
cor MI
M0
M1
M2
M3
M4
M5
M6
ratios
M+H
M+
M−H
M+H2O−CH4
Fumaric acid (−CH4)
M+H = 97.5
M+H2O−CH4 = 2.5
S1_Glc
S2_Glc
S3_Glc
S4_Glc
S5_Glc
S6_Gln
S7_Gln
S8_Gln
S9_Gln
S10_Gln
M−2
M−1
M+0
M+1
M+2
M+3
M+4
M+5
M+6

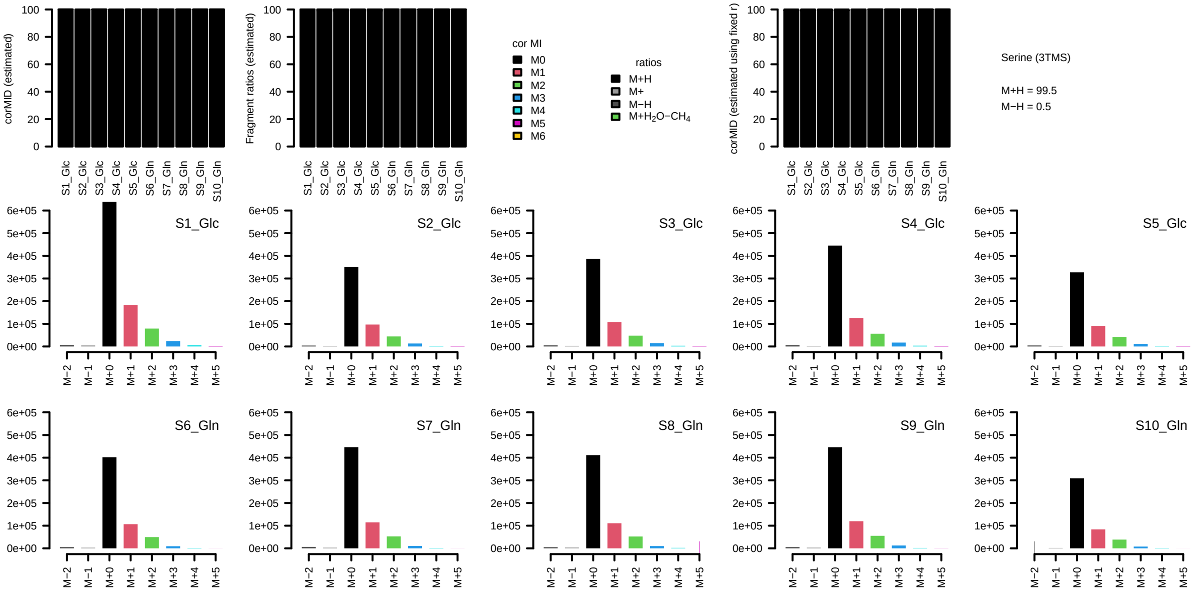
corMID (estimated)
Fragment ratios (estimated)
cor MI
M0
M1
M2
M3
M4
M5
M6
ratios
M+H
M+
M−H
M+H2O−CH4
corMID (estimated using fixed r)
Serine (3TMS)
M+H = 99.5
M−H = 0.5
S1_Glc
S2_Glc
S3_Glc
S4_Glc
S5_Glc
S6_Gln
S7_Gln
S8_Gln
S9_Gln
S10_Gln
M−2
M−1
M+0
M+1
M+2
M+3
M+4
M+5

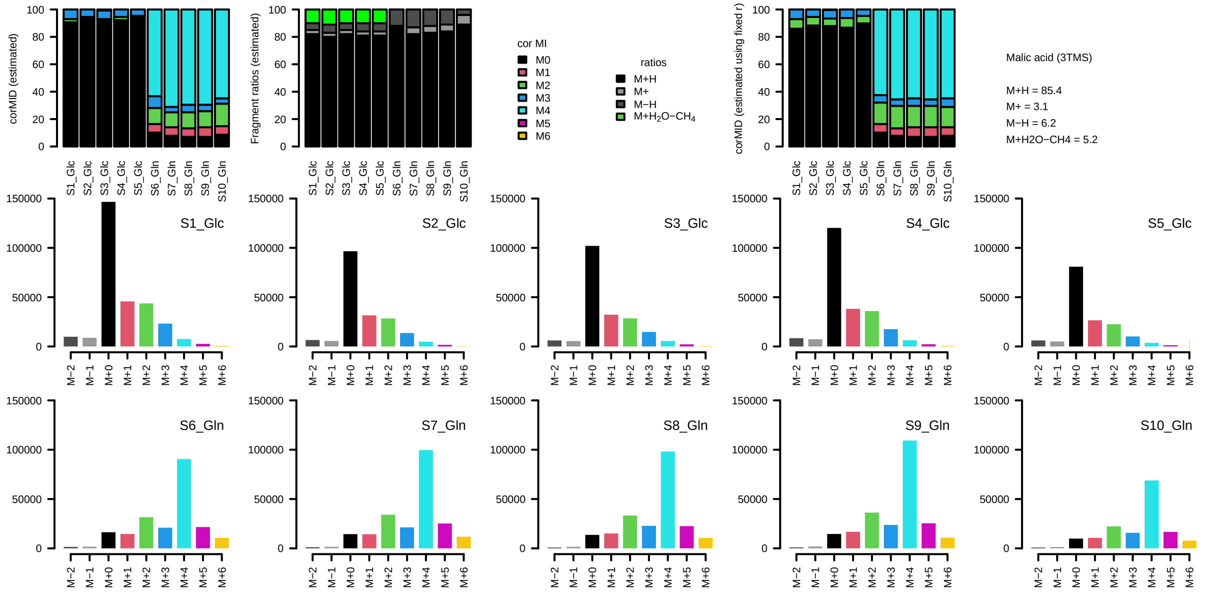
corMID (estimated)
Fragment ratios (estimated)
cor MI
M0
M1
M2
M3
M4
M5
M6
ratios
M+H
M+
M−H
M+H2O−CH4
corMID (estimated using fixed r)
Malic acid (3TMS)
M+H = 85.4
M+ = 3.1
M−H = 6.2
M+H2O−CH4 = 5.2
S1_Glc
S2_Glc
S3_Glc
S4_Glc
S5_Glc
S6_Gln
S7_Gln
S8_Gln
S9_Gln
S10_Gln
M−2
M−1
M+0
M+1
M+2
M+3
M+4
M+5
M+6

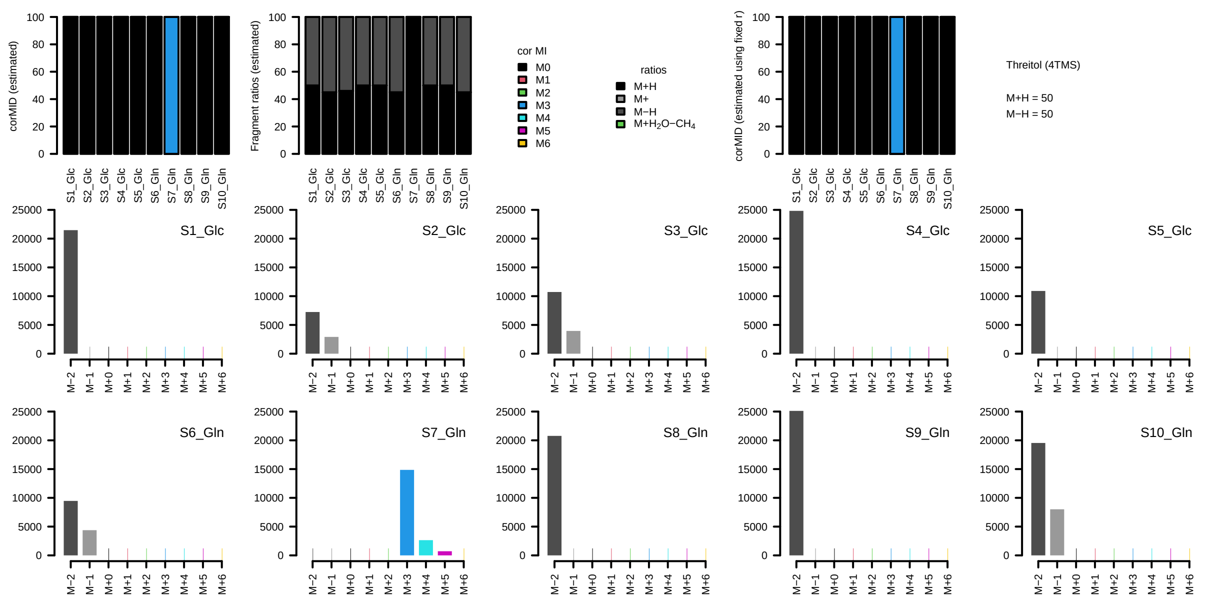
corMID (estimated)
Fragment ratios (estimated)
cor MI
M0
M1
M2
M3
M4
M5
M6
ratios
M+H
M+
M−H
M+H2O−CH4
corMID (estimated using fixed r)
Threitol (4TMS)
M+H = 50
M−H = 50
S1_Glc
S2_Glc
S3_Glc
S4_Glc
S5_Glc
S6_Gln
S7_Gln
S8_Gln
S9_Gln
S10_Gln
M−2
M−1
M+0
M+1
M+2
M+3
M+4
M+5
M+6

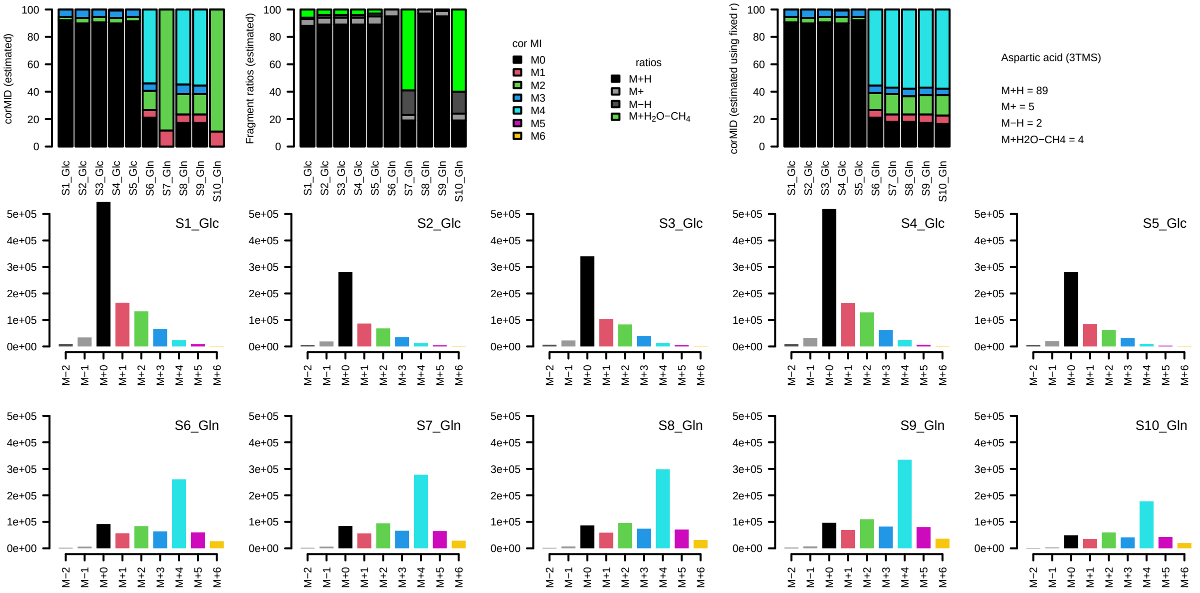

corMID (estimated)
Fragment ratios (estimated)
corMID (estimated using fixed r)
S1_Glc S2_Glc S3_Glc S4_Glc S5_Glc S6_Gln S7_Gln S8_Gln S9_Gln S10_Gln
cor MI
M0
M1
M2
M3
M4
M5
M6
ratios
M+H
M+
M−H
M+H2O−CH4
Aspartic acid (3TMS)
M+H = 89
M+ = 5
M−H = 2
M+H2O−CH4 = 4
S1_Glc
S2_Glc
S3_Glc
S4_Glc
S5_Glc
S6_Gln
S7_Gln
S8_Gln
S9_Gln
S10_Gln
0e+00 1e+05 2e+05 3e+05 4e+05 5e+05
M−2 M−1 M+0 M+1 M+2 M+3 M+4 M+5 M+6

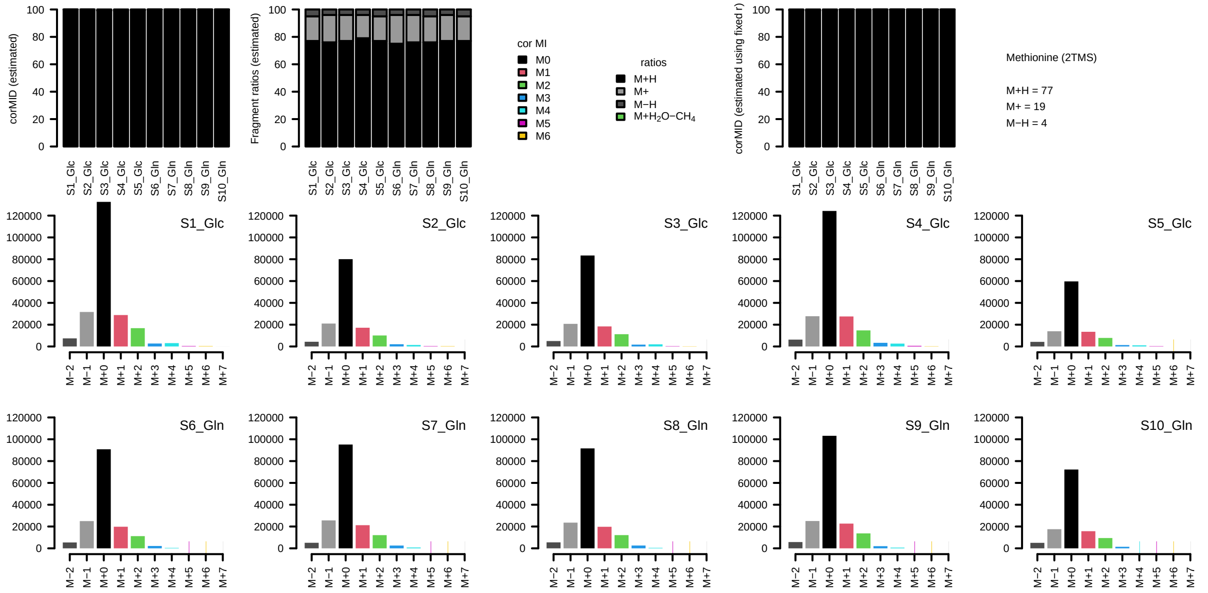
corMID (estimated)
Fragment ratios (estimated)
cor MI
M0
M1
M2
M3
M4
M5
M6
ratios
M+H
M+
M−H
M+H2O−CH4
corMID (estimated using fixed r)
Methionine (2TMS)
M+H = 77
M+ = 19
M−H = 4
S1_Glc
S2_Glc
S3_Glc
S4_Glc
S5_Glc
S6_Gln
S7_Gln
S8_Gln
S9_Gln
S10_Gln
M−2
M−1
M+0
M+1
M+2
M+3
M+4
M+5
M+6
M+7

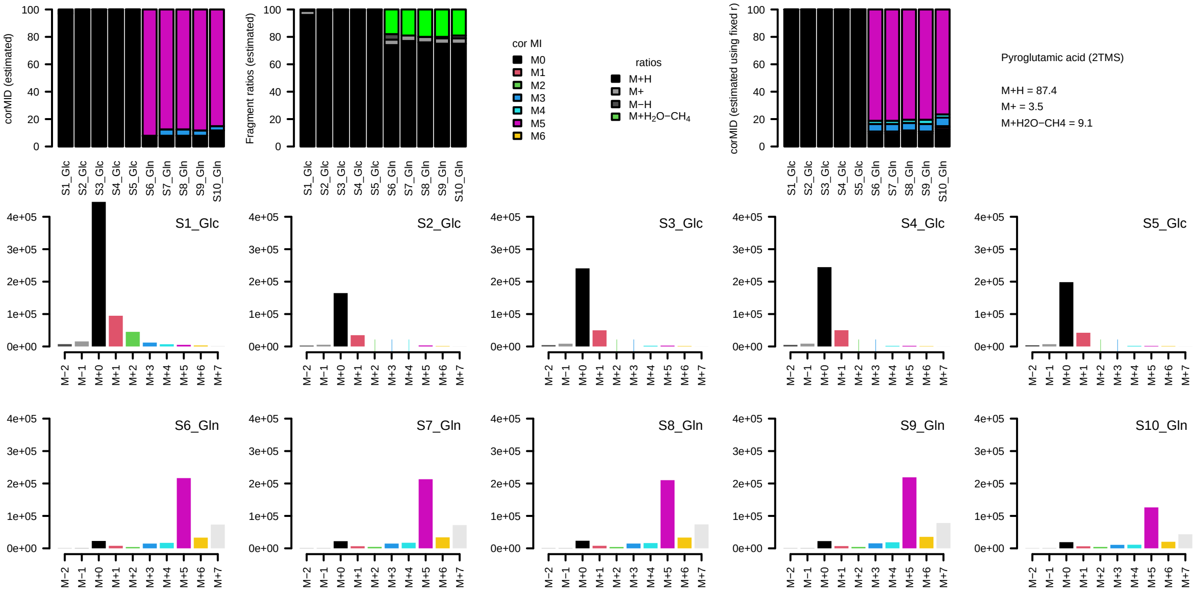
corMID (estimated)
Fragment ratios (estimated)
cor MI
M0
M1
M2
M3
M4
M5
M6
ratios
M+H
M+
M−H
M+H2O−CH4
corMID (estimated using fixed r)
Pyroglutamic acid (2TMS)
M+H = 87.4
M+ = 3.5
M+H2O−CH4 = 9.1
S1_Glc
S2_Glc
S3_Glc
S4_Glc
S5_Glc
S6_Gln
S7_Gln
S8_Gln
S9_Gln
S10_Gln
M−2
M−1
M+0
M+1
M+2
M+3
M+4
M+5
M+6
M+7

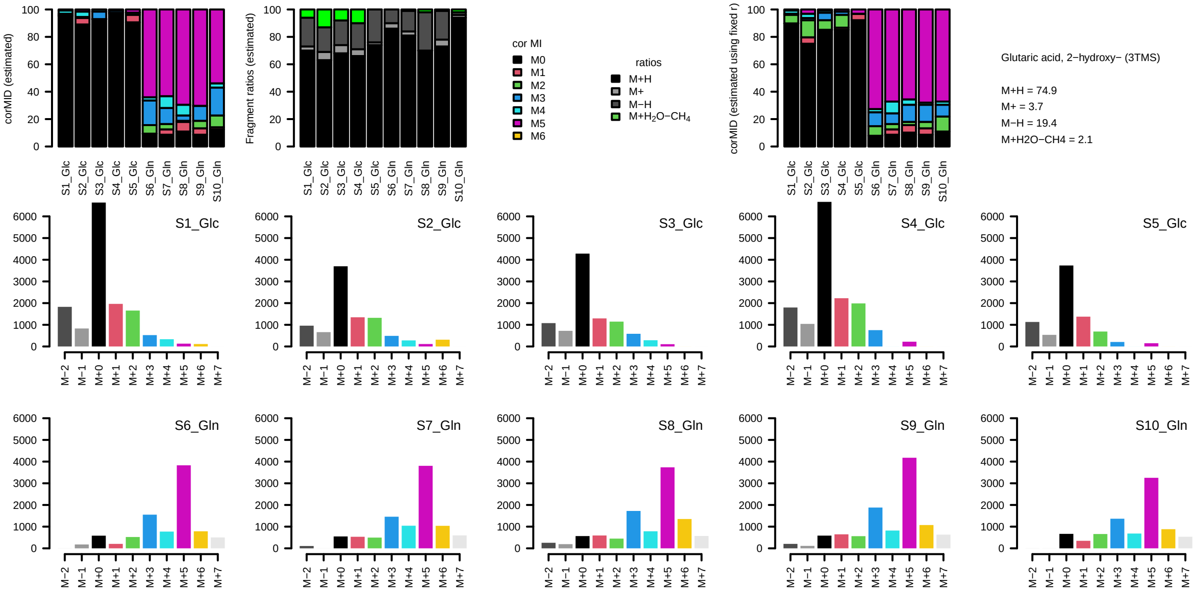

corMID (estimated)
Fragment ratios (estimated)
corMID (estimated using fixed r)
cor MI
M0
M1
M2
M3
M4
M5
M6
ratios
M+H
M+
M−H
M+H2O−CH4
Glutaric acid, 2−hydroxy− (3TMS)
M+H = 74.9
M+ = 3.7
M−H = 19.4
M+H2O−CH4 = 2.1
S1_Glc
S2_Glc
S3_Glc
S4_Glc
S5_Glc
S6_Gln
S7_Gln
S8_Gln
S9_Gln
S10_Gln
M−2 M−1 M+0 M+1 M+2 M+3 M+4 M+5 M+6 M+7

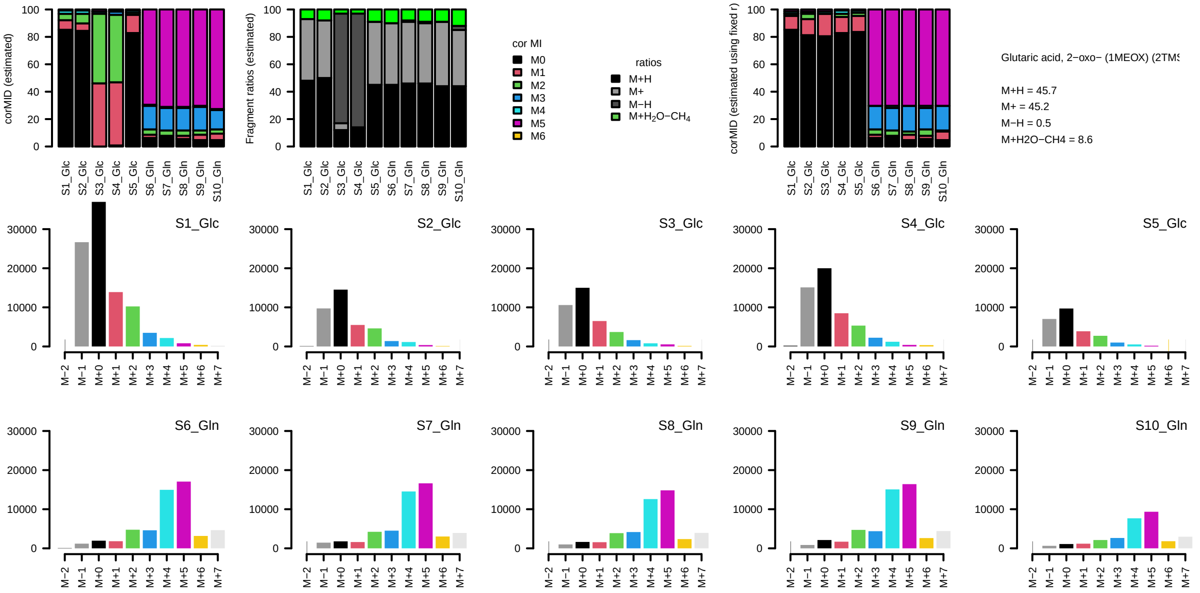
corMID (estimated)
Fragment ratios (estimated)
cor MI
M0
M1
M2
M3
M4
M5
M6
ratios
M+H
M+
M−H
M+H2O−CH4
corMID (estimated using fixed r)
Glutaric acid, 2−oxo− (1MEOX) (2TMS
M+H = 45.7
M+ = 45.2
M−H = 0.5
M+H2O−CH4 = 8.6
S1_Glc
S2_Glc
S3_Glc
S4_Glc
S5_Glc
S6_Gln
S7_Gln
S8_Gln
S9_Gln
S10_Gln
M−2
M−1
M+0
M+1
M+2
M+3
M+4
M+5
M+6
M+7

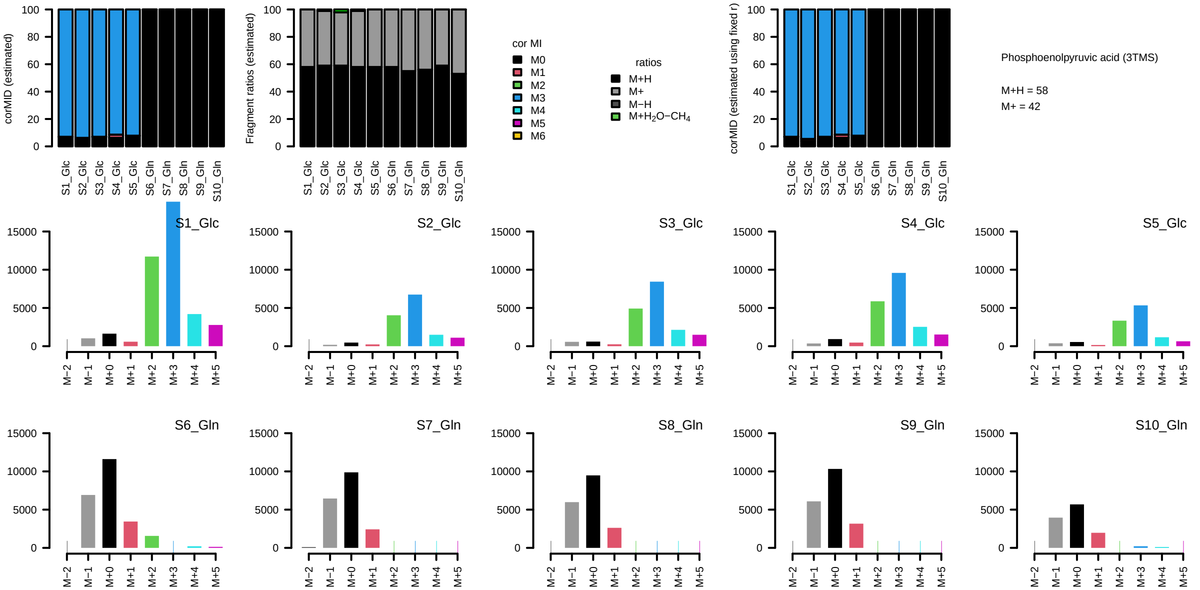

corMID (estimated)
Fragment ratios (estimated)
cor MI
M0
M1
M2
M3
M4
M5
M6
ratios
M+H
M+
M−H
M+H2O−CH4
corMID (estimated using fixed r)
Phosphoenolpyruvic acid (3TMS)
M+H = 58
M+ = 42
S1_Glc
S2_Glc
S3_Glc
S4_Glc
S5_Glc
S6_Gln
S7_Gln
S8_Gln
S9_Gln
S10_Gln
M−2
M−1
M+0
M+1
M+2
M+3
M+4
M+5

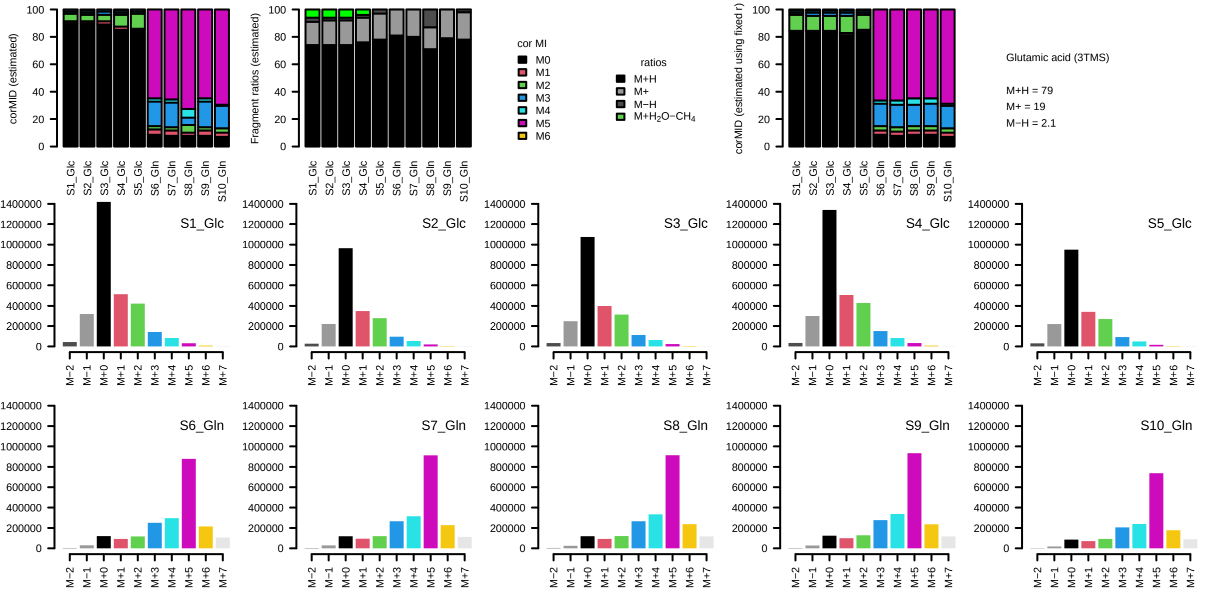

corMID (estimated)
Fragment ratios (estimated)
corMID (estimated using fixed r)
cor MI
M0
M1
M2
M3
M4
M5
M6
ratios
M+H
M+
M−H
M+H_2O−CH_4
Glutamic acid (3TMS)
M+H = 79
M+ = 19
M−H = 2.1
S1_Glc
S2_Glc
S3_Glc
S4_Glc
S5_Glc
S6_Gln
S7_Gln
S8_Gln
S9_Gln
S10_Gln

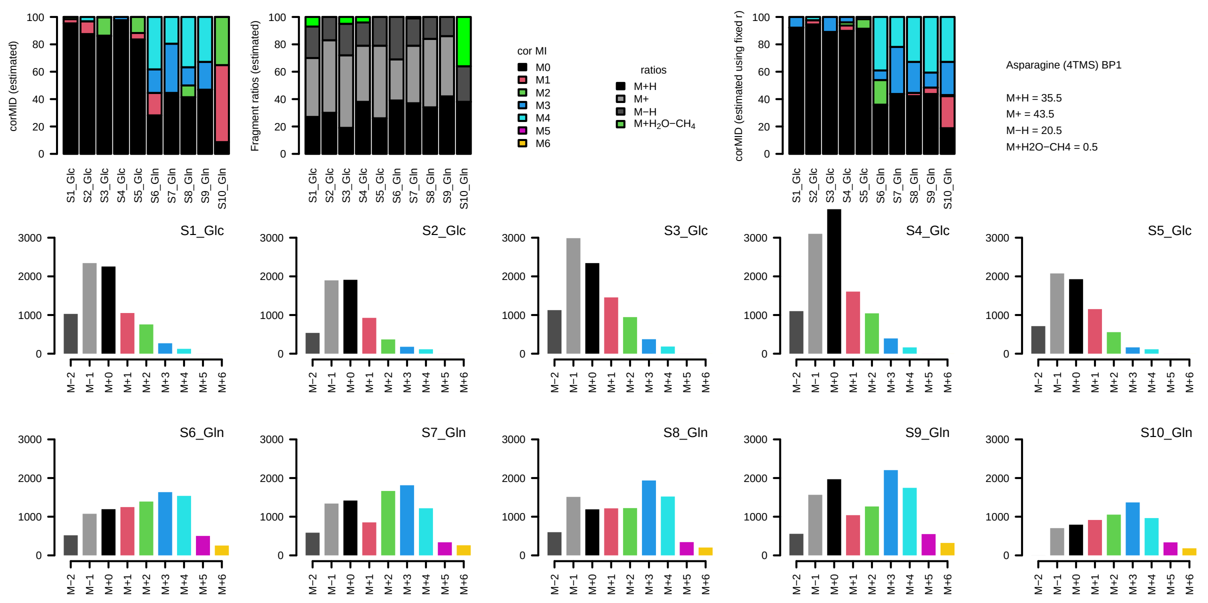
corMID (estimated)
Fragment ratios (estimated)
cor MI
M0
M1
M2
M3
M4
M5
M6
ratios
M+H
M+
M−H
M+H2O−CH4
corMID (estimated using fixed r)
Asparagine (4TMS) BP1
M+H = 35.5
M+ = 43.5
M−H = 20.5
M+H2O−CH4 = 0.5
S1_Glc
S2_Glc
S3_Glc
S4_Glc
S5_Glc
S6_Gln
S7_Gln
S8_Gln
S9_Gln
S10_Gln
M−2
M−1
M+0
M+1
M+2
M+3
M+4
M+5
M+6

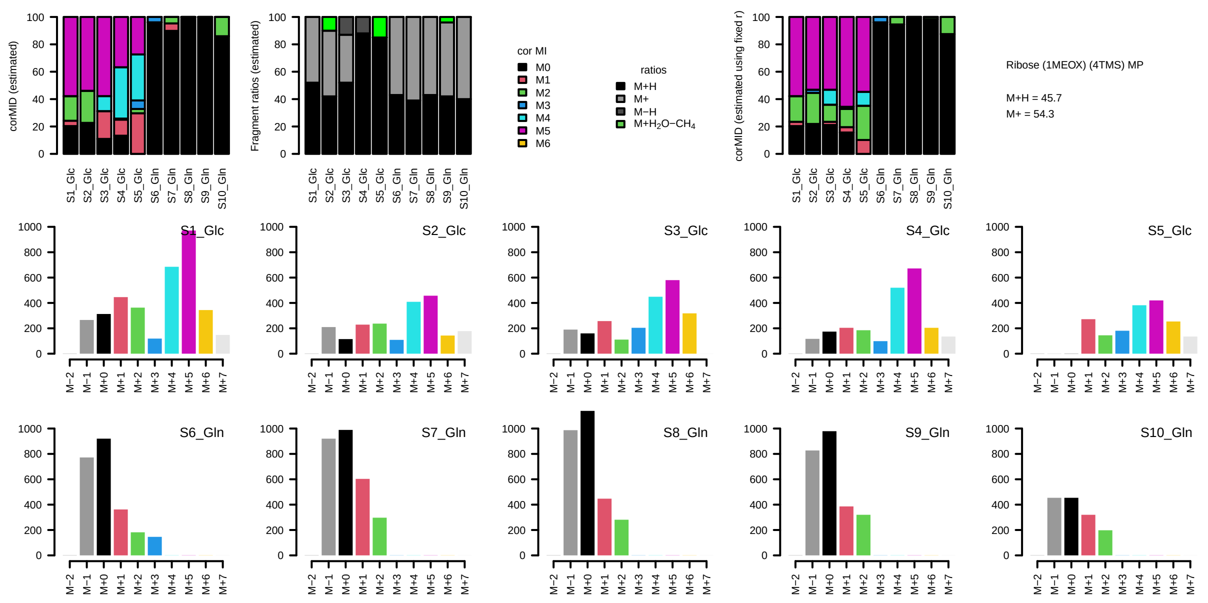
corMID (estimated)
Fragment ratios (estimated)
corMID (estimated using fixed r)
cor MI
M0
M1
M2
M3
M4
M5
M6
ratios
M+H
M+
M−H
M+H_2O−CH_4
Ribose (1MEOX) (4TMS) MP
M+H = 45.7
M+ = 54.3
S1_Glc
S2_Glc
S3_Glc
S4_Glc
S5_Glc
S6_Gln
S7_Gln
S8_Gln
S9_Gln
S10_Gln
M−2
M−1
M+0
M+1
M+2
M+3
M+4
M+5
M+6
M+7

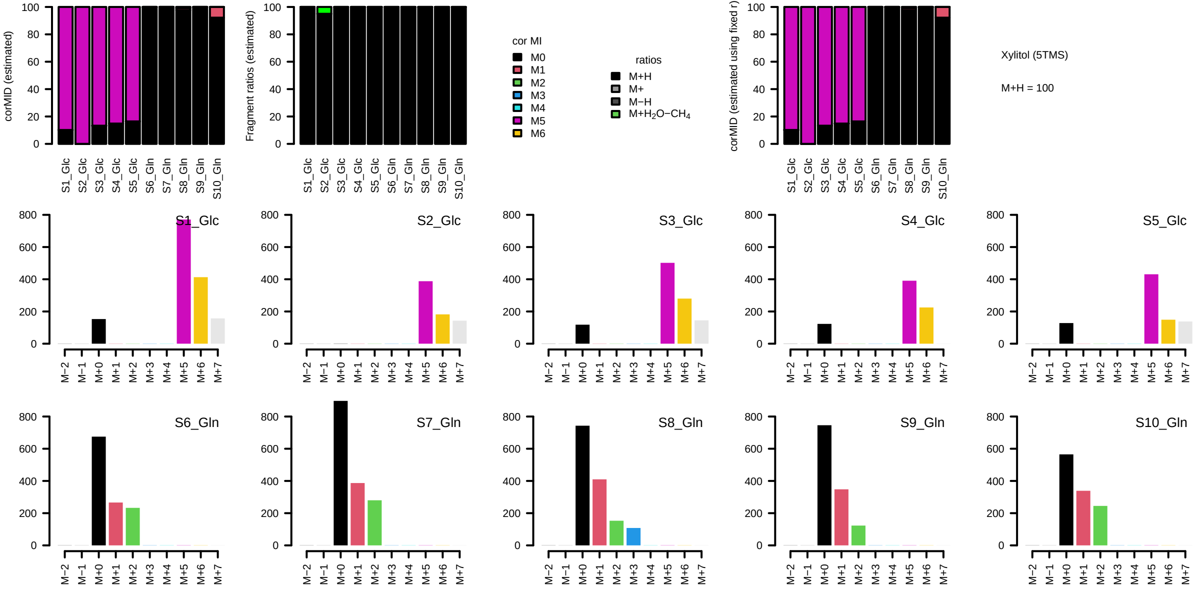

corMID (estimated)
Fragment ratios (estimated)
corMID (estimated using fixed r)
S1_Glc S2_Glc S3_Glc S4_Glc S5_Glc S6_Gln S7_Gln S8_Gln S9_Gln S10_Gln
cor MI
M0
M1
M2
M3
M4
M5
M6
ratios
M+H
M+
M−H
M+H_2O−CH_4
Xylitol (5TMS)
M+H = 100
S1_Glc
S2_Glc
S3_Glc
S4_Glc
S5_Glc
S6_Gln
S7_Gln
S8_Gln
S9_Gln
S10_Gln
M−2 M−1 M+0 M+1 M+2 M+3 M+4 M+5 M+6 M+7

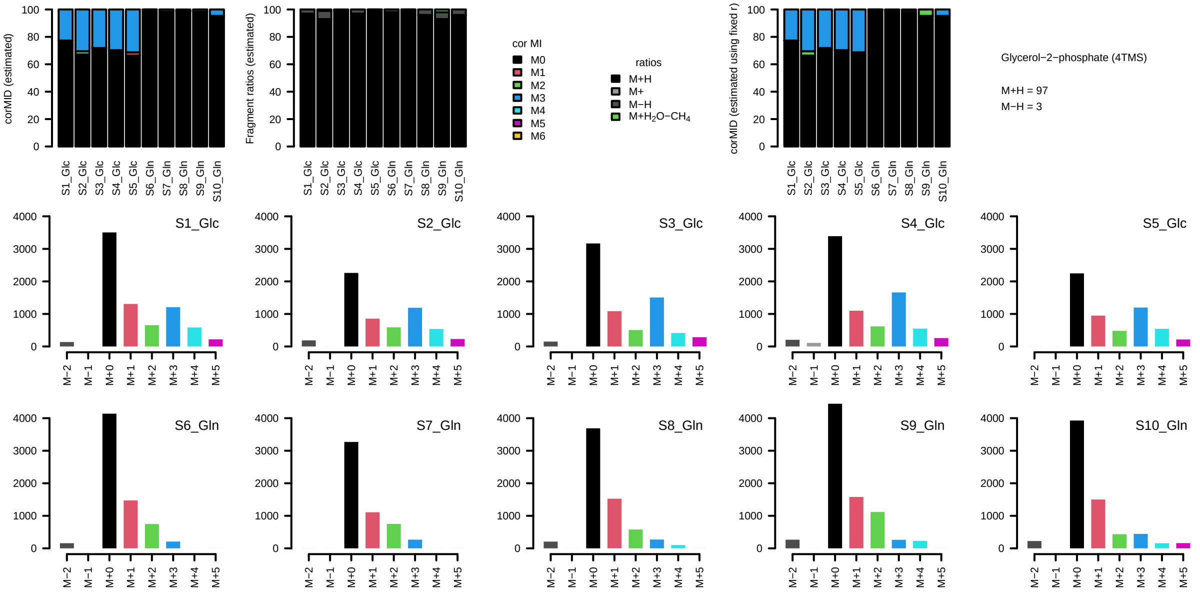
corMID (estimated)
Fragment ratios (estimated)
cor MI
M0
M1
M2
M3
M4
M5
M6
ratios
M+H
M+
M−H
M+H_2O−CH_4
corMID (estimated using fixed r)
Glycerol−2−phosphate (4TMS)
M+H = 97
M−H = 3
S1_Glc
S2_Glc
S3_Glc
S4_Glc
S5_Glc
S6_Gln
S7_Gln
S8_Gln
S9_Gln
S10_Gln
M−2
M−1
M+0
M+1
M+2
M+3
M+4
M+5

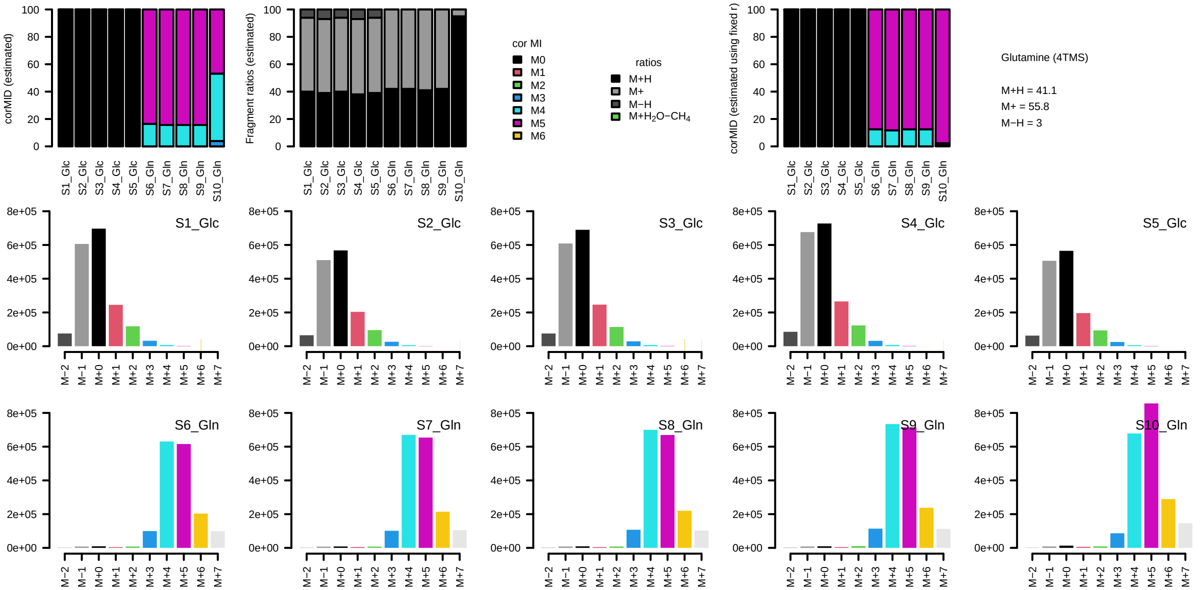
corMID (estimated)
Fragment ratios (estimated)
cor MI
M0
M1
M2
M3
M4
M5
M6
ratios
M+H
M+
M−H
M+H_2O−CH_4
corMID (estimated using fixed r)
Glutamine (4TMS)
M+H = 41.1
M+ = 55.8
M−H = 3
S1_Glc
S2_Glc
S3_Glc
S4_Glc
S5_Glc
S6_Gln
S7_Gln
S8_Gln
S9_Gln
S10_Gln

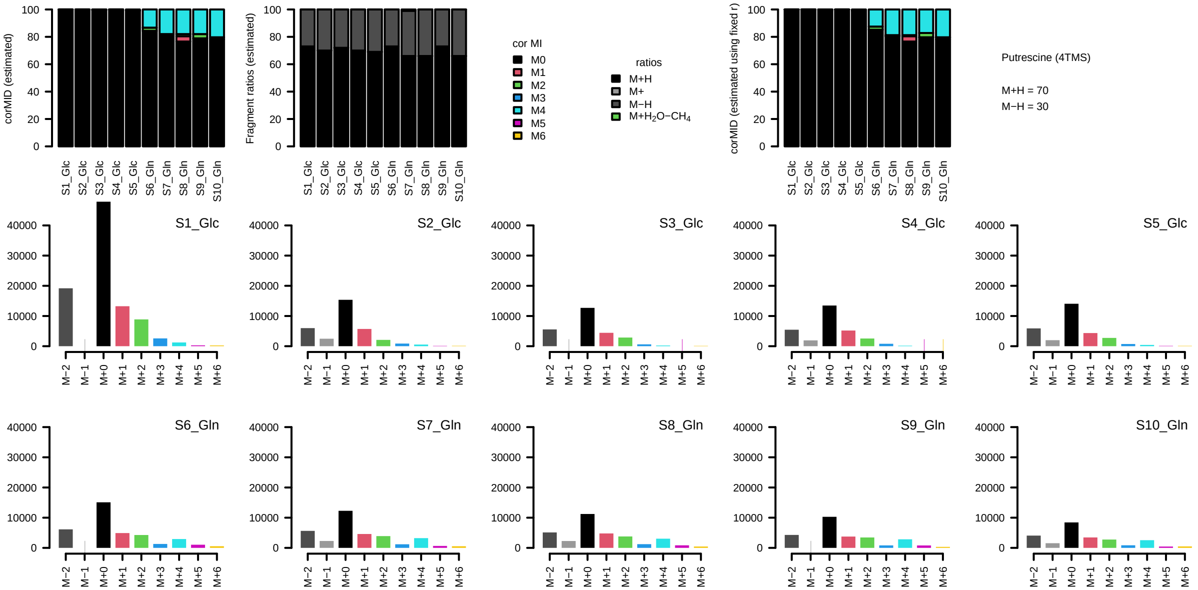
corMID (estimated)
Fragment ratios (estimated)
corMID (estimated using fixed r)
cor MI
M0
M1
M2
M3
M4
M5
M6
ratios
M+H
M+
M−H
M+H2O−CH4
Putrescine (4TMS)
M+H = 70
M−H = 30
S1_Glc
S2_Glc
S3_Glc
S4_Glc
S5_Glc
S6_Gln
S7_Gln
S8_Gln
S9_Gln
S10_Gln
M−2
M−1
M+0
M+1
M+2
M+3
M+4
M+5
M+6

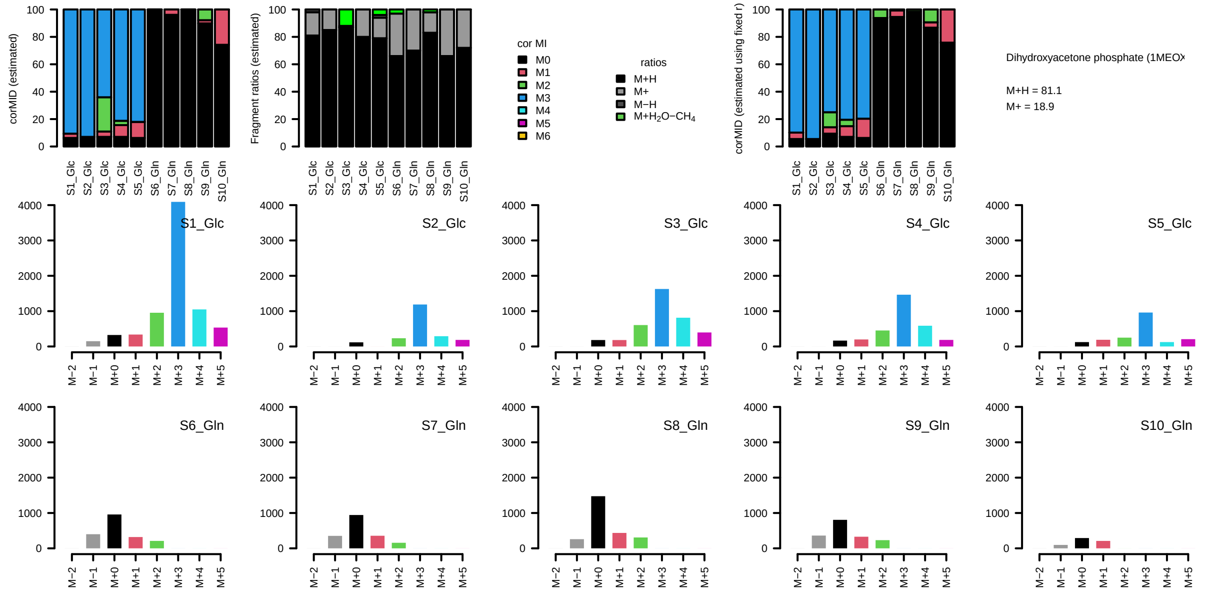

corMID (estimated)
Fragment ratios (estimated)
corMID (estimated using fixed r)
cor MI
M0
M1
M2
M3
M4
M5
M6
ratios
M+H
M+
M−H
M+H2O−CH4
Dihydroxyacetone phosphate (1MEOX
M+H = 81.1
M+ = 18.9
S1_Glc
S2_Glc
S3_Glc
S4_Glc
S5_Glc
S6_Gln
S7_Gln
S8_Gln
S9_Gln
S10_Gln

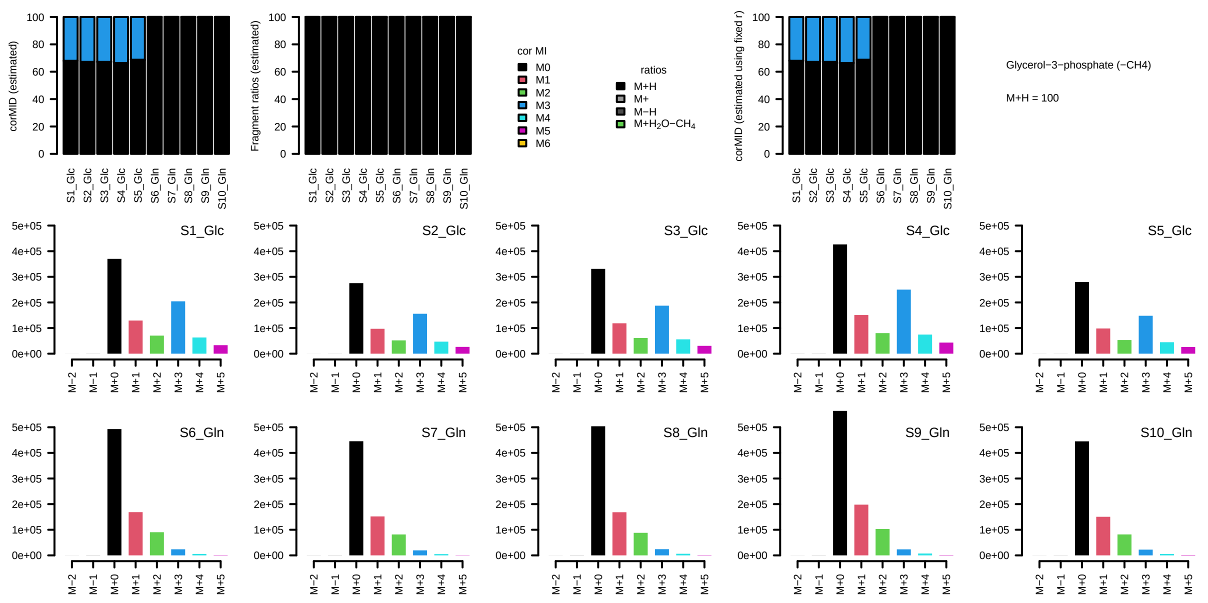
corMID (estimated)
Fragment ratios (estimated)
corMID (estimated using fixed r)
cor MI
M0
M1
M2
M3
M4
M5
M6
ratios
M+H
M+
M−H
M+H_2O−CH_4
Glycerol−3−phosphate (−CH4)
M+H = 100
S1_Glc
S2_Glc
S3_Glc
S4_Glc
S5_Glc
S6_Gln
S7_Gln
S8_Gln
S9_Gln
S10_Gln
M−2
M−1
M+0
M+1
M+2
M+3
M+4
M+5

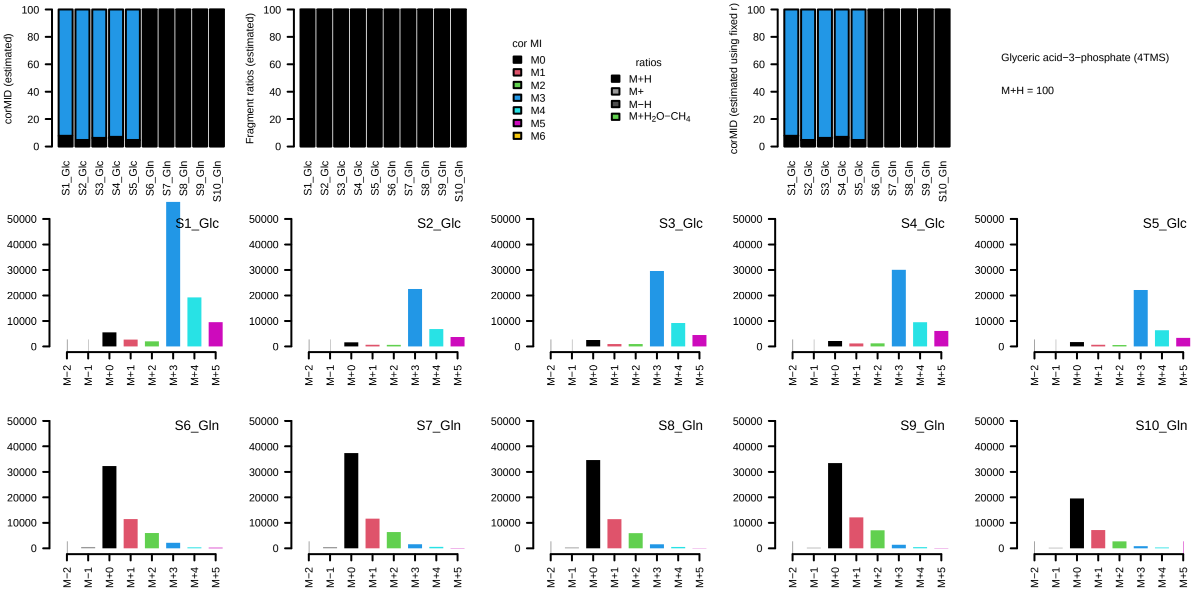

corMID (estimated)
Fragment ratios (estimated)
corMID (estimated using fixed r)
cor MI
M0
M1
M2
M3
M4
M5
M6
ratios
M+H
M+
M−H
M+H2O−CH4
Glyceric acid−3−phosphate (4TMS)
M+H = 100
S1_Glc
S2_Glc
S3_Glc
S4_Glc
S5_Glc
S6_Gln
S7_Gln
S8_Gln
S9_Gln
S10_Gln
M−2
M−1
M+0
M+1
M+2
M+3
M+4
M+5

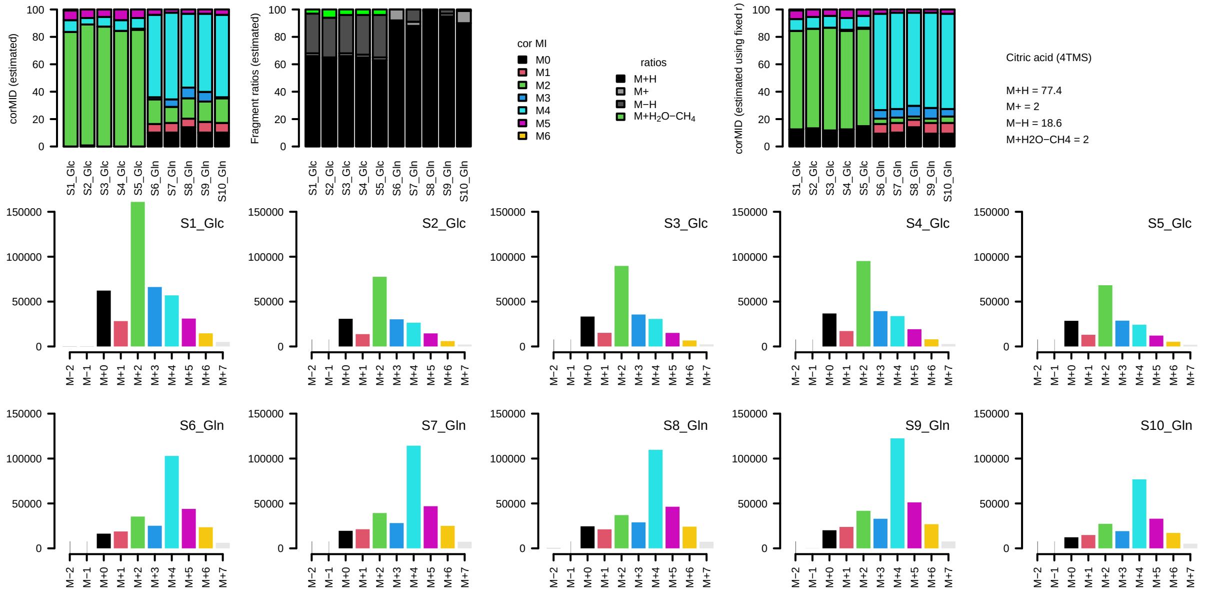
corMID (estimated)
Fragment ratios (estimated)
cor MI
M0
M1
M2
M3
M4
M5
M6
ratios
M+H
M+
M−H
M+H2O−CH4
corMID (estimated using fixed r)
Citric acid (4TMS)
M+H = 77.4
M+ = 2
M−H = 18.6
M+H2O−CH4 = 2
S1_Glc
S2_Glc
S3_Glc
S4_Glc
S5_Glc
S6_Gln
S7_Gln
S8_Gln
S9_Gln
S10_Gln
M−2
M−1
M+0
M+1
M+2
M+3
M+4
M+5
M+6
M+7

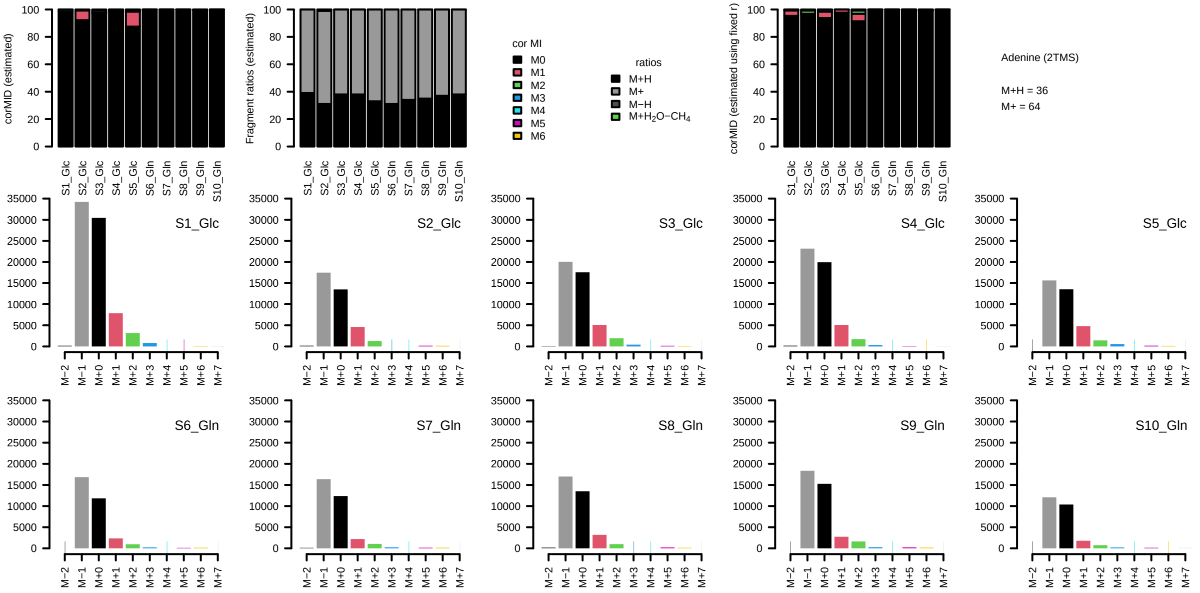

corMID (estimated)
Fragment ratios (estimated)
cor MI
M0
M1
M2
M3
M4
M5
M6
ratios
M+H
M+
M−H
M+H2O−CH4
corMID (estimated using fixed r)
Adenine (2TMS)
M+H = 36
M+ = 64
S1_Glc
S2_Glc
S3_Glc
S4_Glc
S5_Glc
S6_Gln
S7_Gln
S8_Gln
S9_Gln
S10_Gln
M−2
M−1
M+0
M+1
M+2
M+3
M+4
M+5
M+6
M+7

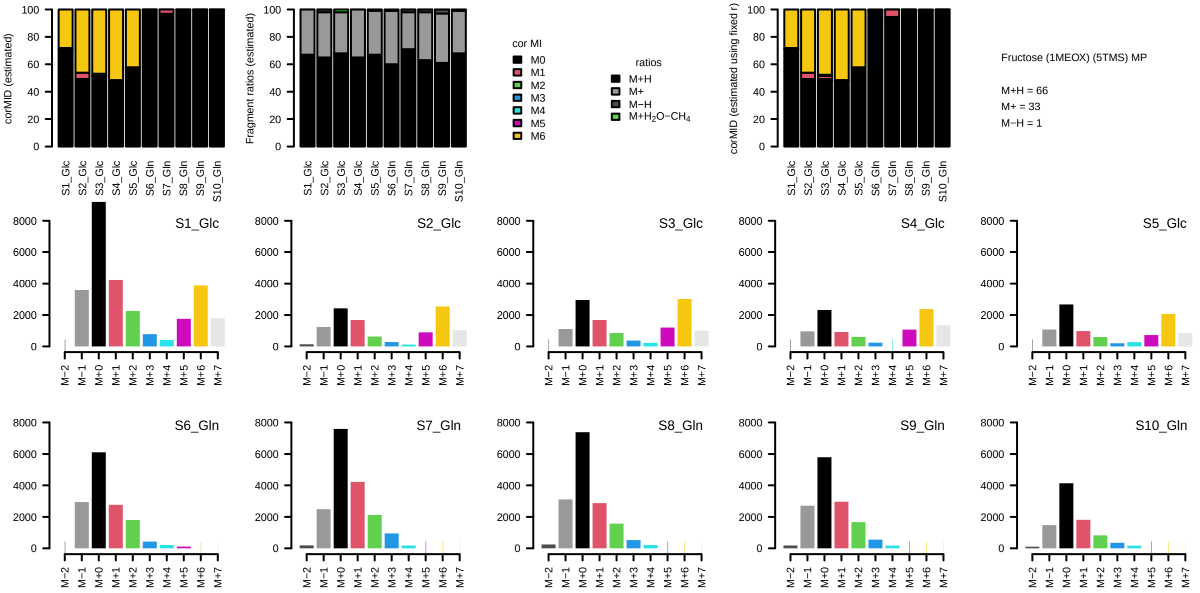
corMID (estimated)
Fragment ratios (estimated)
corMID (estimated using fixed r)
cor MI
M0
M1
M2
M3
M4
M5
M6
ratios
M+H
M+
M−H
M+H2O−CH4
Fructose (1MEOX) (5TMS) MP
M+H = 66
M+ = 33
M−H = 1
S1_Glc
S2_Glc
S3_Glc
S4_Glc
S5_Glc
S6_Gln
S7_Gln
S8_Gln
S9_Gln
S10_Gln
M−2
M−1
M+0
M+1
M+2
M+3
M+4
M+5
M+6
M+7

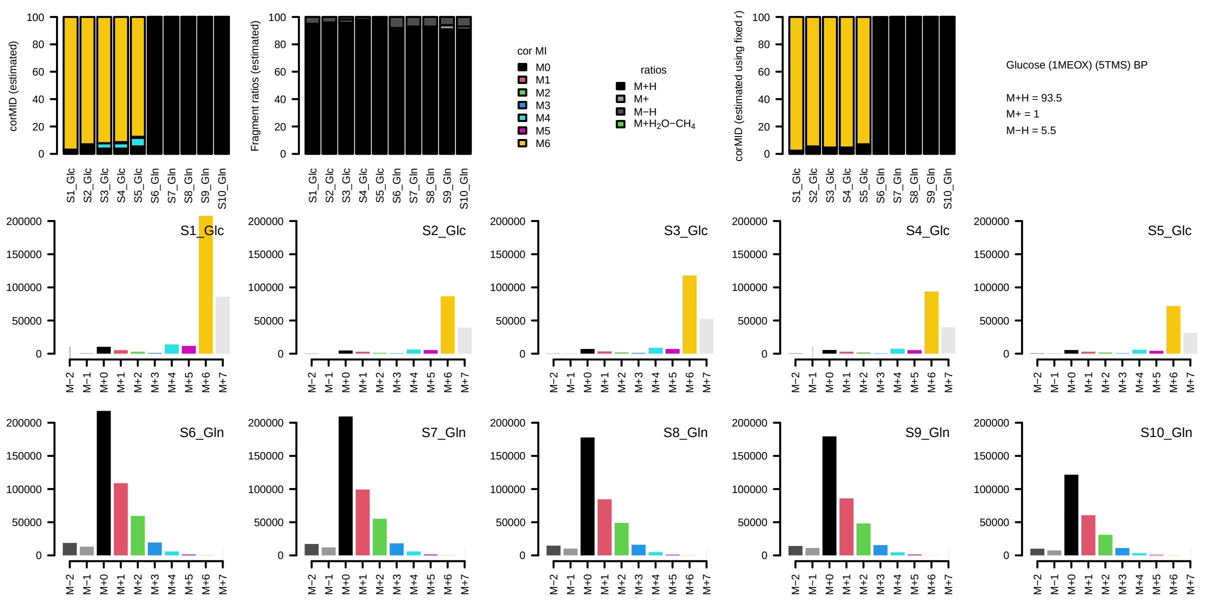

corMID (estimated)
Fragment ratios (estimated)
corMID (estimated using fixed r)
cor MI
M0
M1
M2
M3
M4
M5
M6
ratios
M+H
M+
M−H
M+H2O−CH4
Glucose (1MEOX) (5TMS) BP
M+H = 93.5
M+ = 1
M−H = 5.5
S1_Glc
S2_Glc
S3_Glc
S4_Glc
S5_Glc
S6_Gln
S7_Gln
S8_Gln
S9_Gln
S10_Gln
M−2
M−1
M+0
M+1
M+2
M+3
M+4
M+5
M+6
M+7

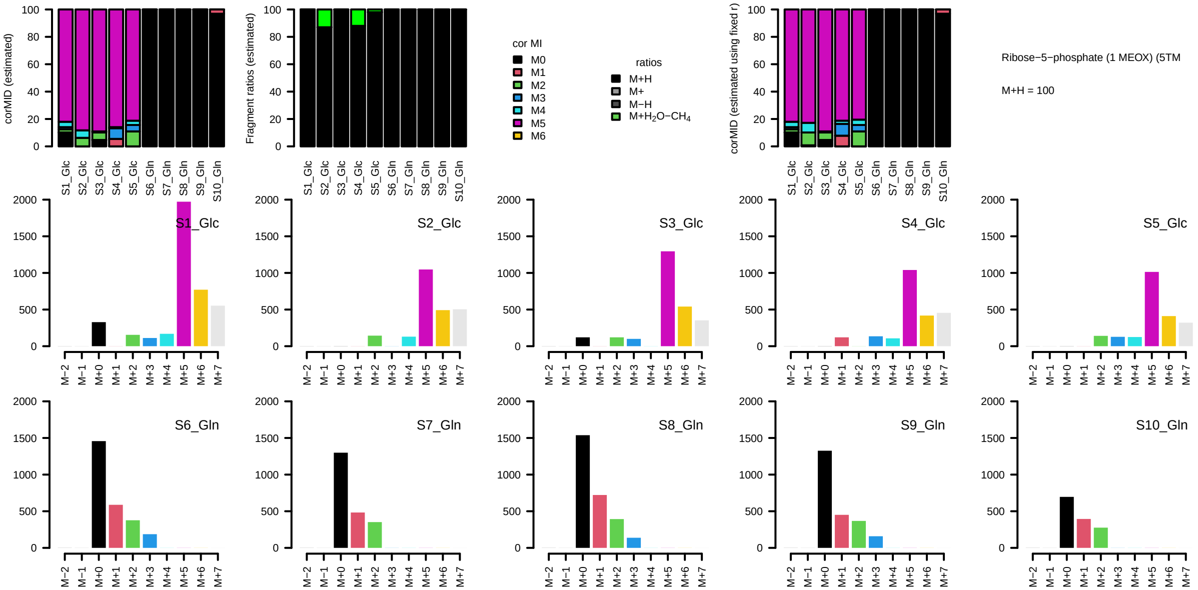
corMID (estimated)
Fragment ratios (estimated)
corMID (estimated using fixed r)
cor MI
M0
M1
M2
M3
M4
M5
M6
ratios
M+H
M+
M−H
M+H_2O−CH_4
Ribose−5−phosphate (1 MEOX) (5TM
M+H = 100
S1_Glc
S2_Glc
S3_Glc
S4_Glc
S5_Glc
S6_Gln
S7_Gln
S8_Gln
S9_Gln
S10_Gln
M−2
M−1
M+0
M+1
M+2
M+3
M+4
M+5
M+6
M+7

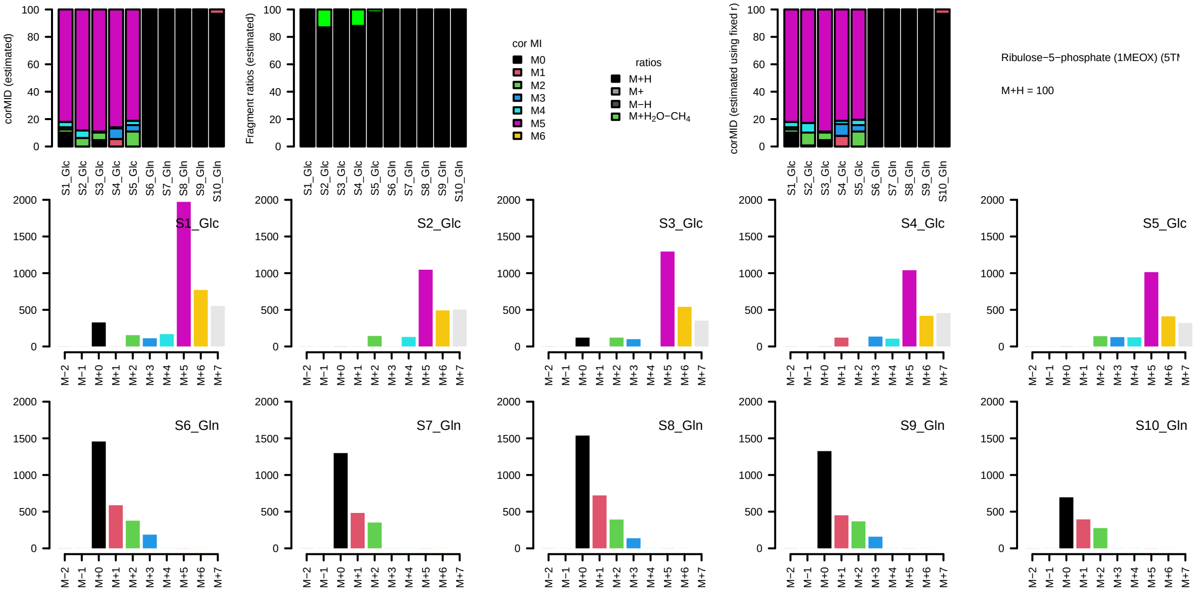

corMID (estimated)
Fragment ratios (estimated)
corMID (estimated using fixed r)
cor MI
M0
M1
M2
M3
M4
M5
M6
ratios
M+H
M+
M−H
M+H2O−CH4
Ribulose−5−phosphate (1MEOX) (5TI
M+H = 100
S1_Glc
S2_Glc
S3_Glc
S4_Glc
S5_Glc
S6_Gln
S7_Gln
S8_Gln
S9_Gln
S10_Gln
M−2
M−1
M+0
M+1
M+2
M+3
M+4
M+5
M+6
M+7

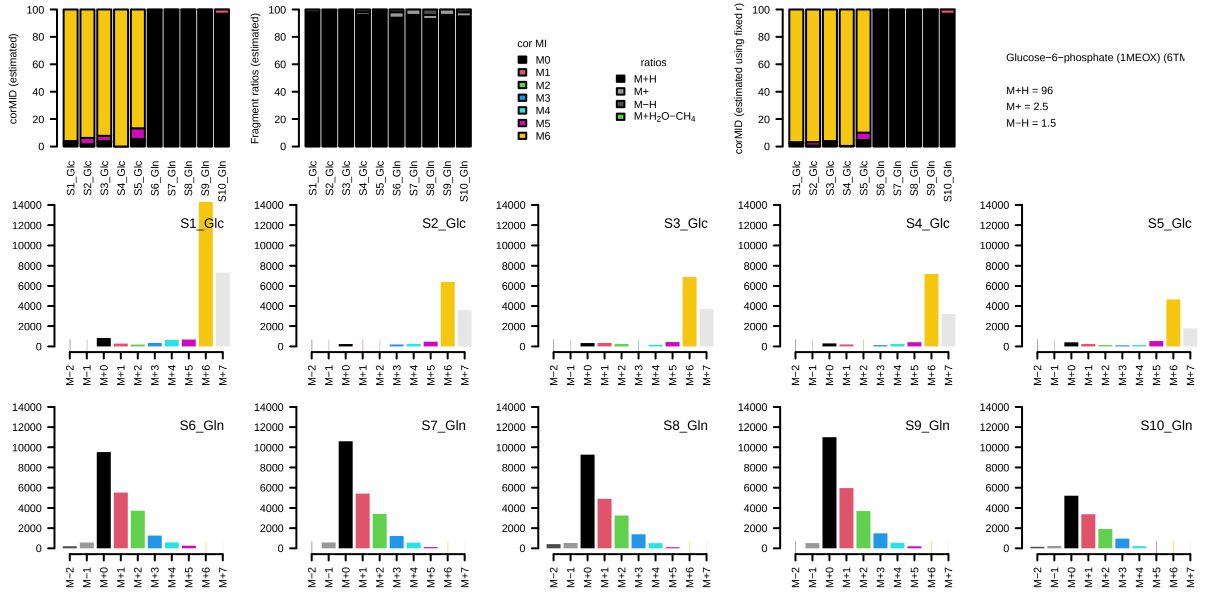

corMID (estimated)
Fragment ratios (estimated)
corMID (estimated using fixed r)
cor MI
M0
M1
M2
M3
M4
M5
M6
ratios
M+H
M+
M−H
M+H$_2$O−CH$_4$
Glucose−6−phosphate (1MEOX) (6TM
M+H = 96
M+ = 2.5
M−H = 1.5
S1_Glc
S2_Glc
S3_Glc
S4_Glc
S5_Glc
S6_Gln
S7_Gln
S8_Gln
S9_Gln
S10_Gln
M−2
M−1
M+0
M+1
M+2
M+3
M+4
M+5
M+6
M+7

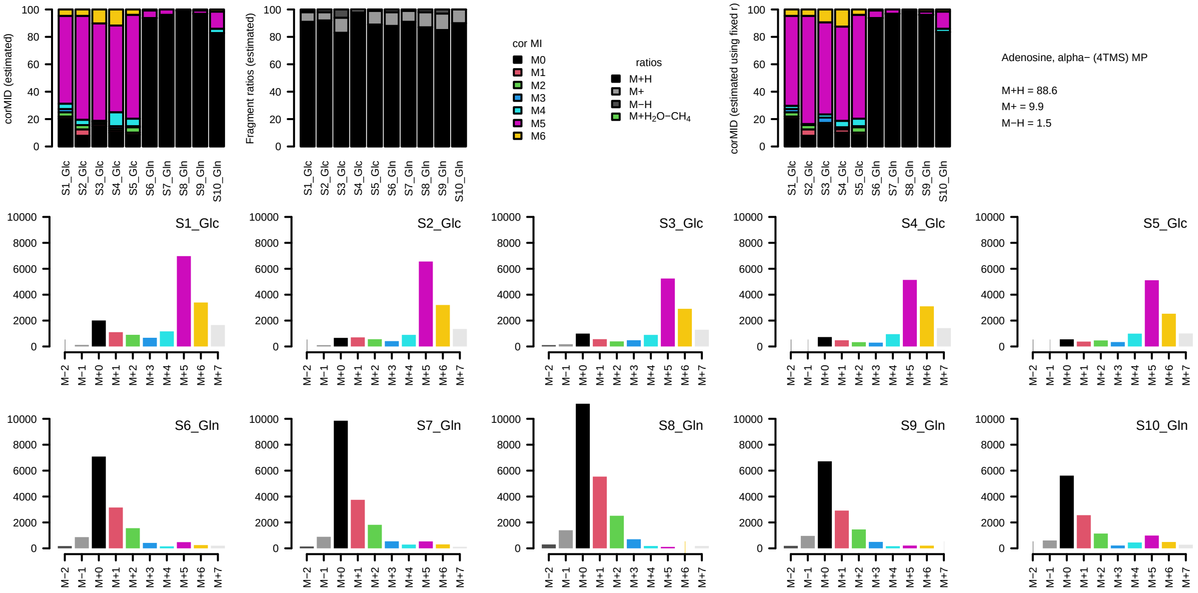
corMID (estimated)
Fragment ratios (estimated)
corMID (estimated using fixed r)
cor MI
M0
M1
M2
M3
M4
M5
M6
ratios
M+H
M+
M−H
M+H2O−CH4
Adenosine, alpha− (4TMS) MP
M+H = 88.6
M+ = 9.9
M−H = 1.5
S1_Glc
S2_Glc
S3_Glc
S4_Glc
S5_Glc
S6_Gln
S7_Gln
S8_Gln
S9_Gln
S10_Gln
M−2
M−1
M+0
M+1
M+2
M+3
M+4
M+5
M+6
M+7
